# Hypoxia-mediated stabilization of HIF1A in prostatic intraepithelial neoplasia promotes cell plasticity and malignant progression

**DOI:** 10.1126/sciadv.abo2295

**Published:** 2022-07-22

**Authors:** Mohamed A. Abu el Maaty, Julie Terzic, Céline Keime, Daniela Rovito, Régis Lutzing, Darya Yanushko, Maxime Parisotto, Elise Grelet, Izzie Jacques Namer, Véronique Lindner, Gilles Laverny, Daniel Metzger

**Affiliations:** ^1^ Institut de Génétique et de Biologie Moléculaire et Cellulaire, Illkirch, France.; ^2^ Centre National de la Recherche Scientifique (CNRS), UMR7104, Illkirch, France.; ^3^ Institut National de la Santé et de la Recherche Médicale (INSERM), U1258, Illkirch, France.; ^4^ Université de Strasbourg, Strasbourg, France.; ^5^ ICube, CNRS, UMR 7357, Strasbourg, France.; ^6^ Département de Pathologie, Les Hôpitaux Universitaires de Strasbourg, Strasbourg, France.

## Abstract

Prostate cancer (PCa) is a leading cause of cancer-related deaths. The slow evolution of precancerous lesions to malignant tumors provides a broad time frame for preventing PCa. To characterize prostatic intraepithelial neoplasia (PIN) progression, we conducted longitudinal studies on Pten^(i)pe−/−^ mice that recapitulate prostate carcinogenesis in humans. We found that early PINs are hypoxic and that hypoxia-inducible factor 1 alpha (HIF1A) signaling is activated in luminal cells, thus enhancing malignant progression. Luminal HIF1A dampens immune surveillance and drives luminal plasticity, leading to the emergence of cells that overexpress Transglutaminase 2 (TGM2) and have impaired androgen signaling. Elevated TGM2 levels in patients with PCa are associated with shortened progression-free survival after prostatectomy. Last, we show that pharmacologically inhibiting HIF1A impairs cell proliferation and induces apoptosis in PINs. Therefore, our study demonstrates that HIF1A is a target for PCa prevention and that TGM2 is a promising prognostic biomarker of early relapse after prostatectomy.

## Teaser

Activated HIF1A signaling in prostatic premalignant lesions is a target for prostate cancer prevention.

## INTRODUCTION

Prostate cancer (PCa) is the second most commonly occurring cancer in men worldwide and one of the leading causes of cancer-related deaths (*1*). Patients with localized PCa are managed either by active surveillance, radiotherapy, or radical prostatectomy, with or without androgen deprivation (*2*, *3*). However, current treatments are associated with side effects such as urinary incontinence and erectile dysfunction, which negatively affect patients’ quality of life (*2*). Moreover, widespread prostate-specific antigen (PSA) screening for the early detection of PCa has been linked to overdiagnosis and overtreatment, whereas no biomarker is available to discern between tumors that will remain indolent and those that will progress (*2*). Furthermore, current therapies for metastatic PCa are also associated with major side effects and ultimately fail. Hence, preventing disease emergence and identifying prognostic markers are needed to improve the management of PCa.

Prostatic intraepithelial neoplasia (PIN) are premalignant lesions that are precursors of PCa (*2*). A number of processes, including cellular senescence and oxidative stress (*4*), as well as alterations in the microenvironment, are associated with the progression of PINs to adenocarcinoma, which can take decades. However, no therapeutic modality has so far been identified for the treatment of PINs. Inhibitors of 5α-reductase such as finasteride, which limit androgen receptor (AR) signaling by impairing the conversion of testosterone to dihydrotestosterone, have been investigated in the context of PCa prevention (*5*). However, they did not reduce the risk of developing high-grade tumors and thus were not approved (*5*). Therefore, deciphering the molecular events supporting PIN progression is required to develop new diagnostic tools and effective chemopreventive strategies.

The rapid proliferation of cancer cells coupled with insufficient vascularization induces solid tumor hypoxia (*6*). Hypoxia-inducible factors (HIFs) are critical mediators of cellular response to hypoxia. HIFs are transcriptional heterodimers comprising an oxygen-labile alpha (HIFA) subunit and a stable beta subunit (*7*–*9*). Under hypoxic conditions, HIFA is stabilized and regulates the expression of genes encoding proteins involved in angiogenesis, erythropoiesis, energy metabolism, cell growth, and survival (*7*–*9*). HIF1A expression has been shown to be elevated in a number of human cancers (*7*–*10*). Hypoxic regions have been found in localized prostate adenocarcinoma (*11*, *12*), and elevated expression of hypoxia markers, including HIF1A and vascular endothelial growth factor (VEGF), have been shown to identify patients with PCa with elevated risk of biochemical recurrence (*13*). Although the up-regulation of HIF1A has been previously reported in high-grade PINs (*14*), the presence of hypoxia and the functional relevance of HIF1A in these lesions have not been demonstrated. Furthermore, whether HIF1A plays a role in PIN progression is unknown.

We found that early PIN lesions in Pten^(i)pe−/−^ mice, which harbor a prostatic luminal epithelial cell–specific deletion of the tumor suppressor *Pten* at adulthood (*15*), are hypoxic and have elevated HIF1A expression. Moreover, we identified HIF1A as a key driver of malignant progression in prostatic lesions, and Transglutaminase 2 (TGM2), the expression of which correlates with HIF1A signaling in mice and humans, as a potential biomarker of early relapse after prostatectomy in patients with PCa.

## RESULTS

### HIF1A signaling is activated in PINs and promotes their survival and progression

**Fig. 1.**
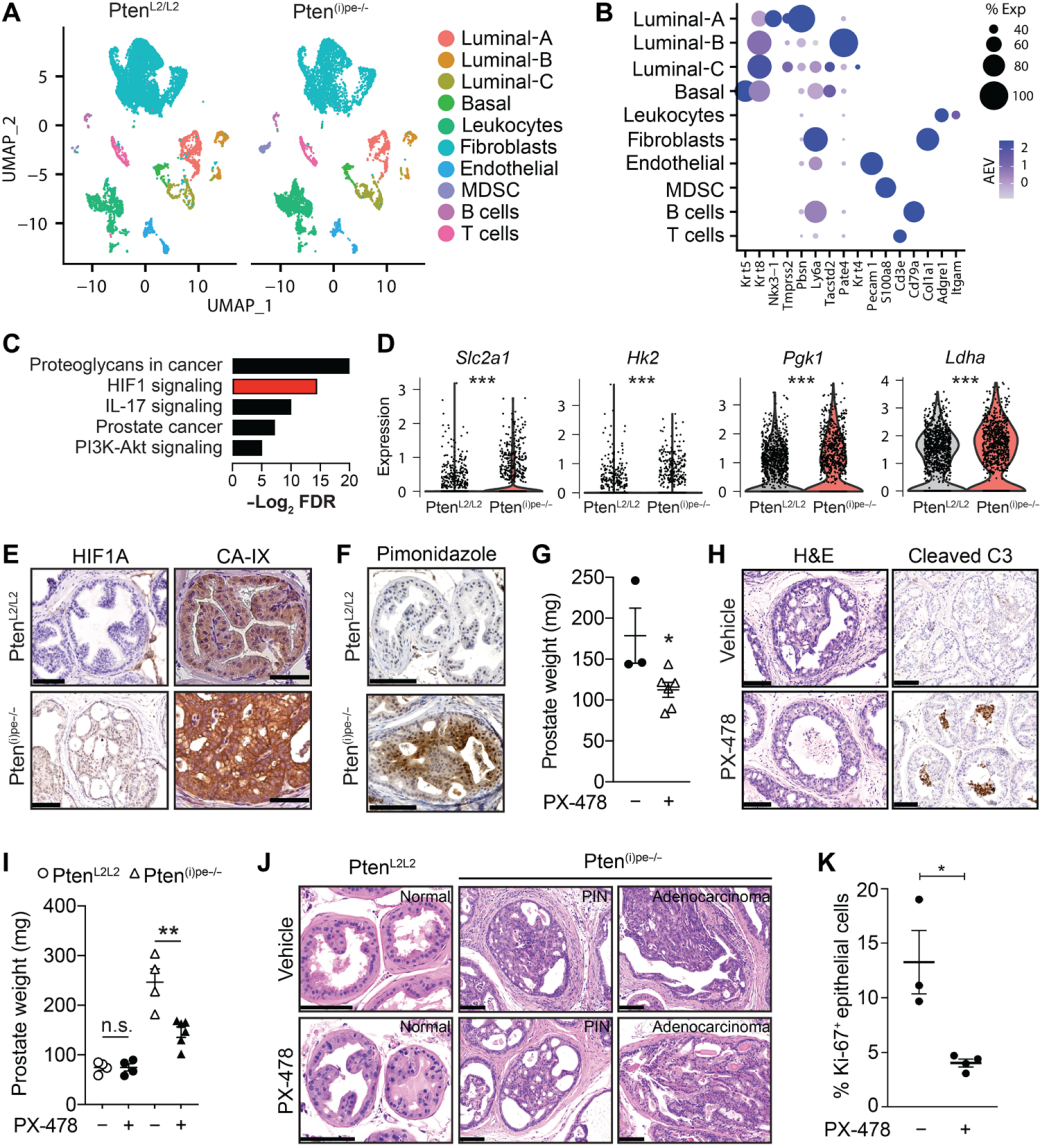
Activation of HIF1A signaling in early prostatic lesions and its pharmacological targeting. (**A**) UMAP of unsupervised clustering of prostatic cells from Pten^L2/L2^ and Pten^(i)pe−/−^ mice, 3 months AGI, and (**B**) dot plot depicting cell lineage–specific markers. (**C**) KEGG pathway analysis of genes up-regulated in Pten-null prostatic luminal cells. (**D**) Violin plots depicting the levels of HIF1A signaling–related transcripts in luminal-A and -C cells. ***P < 0.001, Wilcoxon rank sum test. (**E**) Representative immunohistochemical (IHC) staining of HIF1A and carbonic anhydrase 9 (CA-IX) in sections of dorsolateral prostate (DLP) of Pten^L2/L2^ and Pten^(i)pe−/−^ mice 3 months AGI. *n* = 3 mice per condition. Scale bars, 100 μm. (**F**) Pimonidazole IHC staining in the DLP of Pten^L2/L2^ and Pten^(i)pe−/−^ mice 3 months AGI. *n* = 3 mice per condition. Scale bars, 100 μm. Prostate weight (**G**) and representative H&E staining (**H**, left) and cleaved caspase 3 (C3) IHC staining (**H**, right) of Pten^(i)pe−/−^ mice, 3 months AGI, treated for 5 days with vehicle (*n* = 3) or PX-478 (*n* = 6). *P < 0.05, two-tailed *t* test. Scale bars, 100 μm. Prostate weight (**I**) and H&E-stained DLP (**J**) of Pten^L2/L2^ (*n* = 4 per condition) and Pten^(i)pe−/−^ (*n* = 6 per condition) mice (10 months AGI) treated for 28 days with vehicle or PX-478. n.s., not significant, *P* ≥ 0.05; **P < 0.01, two-tailed *t* test. Scale bars, 100 μm. (**K**) Quantification of Ki-67–positive epithelial cells in the DLP of vehicle- (*n* = 3) and PX-478–treated (*n* = 4) Pten^(i)pe−/−^ mice, 10 months AGI. Five to 10 images per prostatic section were analyzed. *P < 0.05, two-tailed *t* test.

Pten^(i)pe−/−^ mice develop PINs between 1 and 3 months after gene inactivation (AGI) (early lesions), which enter a latency phase until 9 months AGI and evolve into adenocarcinoma between 9 and 12 months AGI (*15*, *16*). To elucidate the transcriptomic changes that occur in various cell types of early PIN lesions, we performed droplet-based single-cell RNA-sequencing (scRNA-seq) on 9956 and 7122 cells isolated from prostates of Pten^L2/L2^ (control) and Pten^(i)pe−/−^ mice, respectively, 3 months AGI. On the basis of the unbiased analysis of the 16,686 cells, 25 clusters were identified, which we broadly classified into epithelial cells (*Epcam*; clusters 5, 11, 12, 14, 15, 18, 20, and 24) and mesenchymal cells, the latter comprising leukocytic (*Ptprc*; clusters 4, 8, 9, 10, 17, 21, 22, and 23) and nonleukocytic (*Vim*; clusters 0, 1, 2, 3, 6, 7, 13, 16, and 19) cells (fig. S1, A and B, and table S1). The leukocytic clusters included T (*Cd3e*) and B (*Cd79a*) lymphocytes, myeloid-derived suppressor cells (MDSCs; *S100a8*), and other immune cells expressing the macrophage marker *Adgre1* (Fig. 1, A and B). Nonleukocytic mesenchymal cells included endothelial cells (*Pecam1*) and stromal fibroblasts (*Col1a1*) (Fig. 1, A and B). Epithelial clusters comprised basal cells (*Krt5*), as well as three luminal subsets (*Krt8*), including a cluster expressing elevated levels of AR target genes such as *Pbsn*, *Nkx3-1*, and *Tmprss2*; a cluster expressing the epididymal marker *Pate4*, indicating the nonprostatic origin of these cells; and a cluster expressing *Krt4* and *Tacstd2* [TROP2 (Trophoblast cell-surface antigen 2)], termed luminal-A, -B, and -C, respectively (Fig. 1, A and B) (*17*). Flow cytometry and immunostaining studies revealed that luminal-C cells are the major epithelial subset in Pten^(i)pe−/−^ prostates at 3 months AGI but are only a minor population in control ones (fig. S1, C and D). Moreover, despite expressing lower levels of canonical AR target genes (Fig. 1B), strong nuclear AR staining was observed in TROP2-positive luminal cells of Pten^(i)pe−/−^ mice (fig. S1E).

Comparison of the transcriptomes of luminal cells of Pten^(i)pe−/−^ and control mice identified more than 600 differentially expressed genes (DEGs) (table S2). As expected, the Kyoto Encyclopedia of Genes and Genomes (KEGG) pathway analysis of genes up-regulated in prostatic luminal cells of Pten^(i)pe−/−^ mice revealed a number of cancer-associated pathways, such as “proteoglycans in cancer,” “PCa,” and “PI3K-Akt signaling” (Fig. 1C). HIF1 signaling was among the top 10 identified pathways (Fig. 1C and table S3), and transcript levels of a number of HIF1A target genes (e.g., *Slc2a1*, *Hk2*, *Pgk1*, and *Ldha*) were higher in luminal cells of Pten^(i)pe−/−^ mice than in those of control mice (Fig. 1D). Furthermore, immunohistochemical analysis at 3 months AGI on independent samples revealed elevated HIF1A protein levels in epithelial cells of Pten^(i)pe−/−^ PINs compared to those of control mice, as well as of carbonic anhydrase IX (CA-IX), which is encoded by the HIF1A target gene *Car9* (*9*) (Fig. 1E). Moreover, Western blot analysis demonstrated elevated levels of the HIF1A target genes *Hk2*, *Eno1*, *Ldha*, and *Gapdh* in prostates of Pten^(i)pe−/−^ mice 3 months AGI compared to control mice (fig. S1F). Together, these results demonstrate that HIF1A levels are increased in early PIN lesions and that HIF1A signaling is activated. Of note, our scRNA-seq analyses showed that unlike their luminal counterparts, HIF1A signaling was not enhanced in basal cells of Pten^(i)pe−/−^ mice (tables S4 and S5).

As hypoxia leads to HIF1A protein stabilization, we determined whether these early lesions were hypoxic. We therefore administered pimonidazole, an agent that is reductively activated under hypoxic conditions and subsequently forms protein adducts in tissues, to control and Pten^(i)pe−/−^ mice 3 months AGI. Immunohistochemical analyses revealed that most PIN cells were pimonidazole positive, in contrast to epithelial cells of control mice (Fig. 1F). Remarkably, a large number of epithelial cells of Pten^(i)pe−/−^ prostates were also pimonidazole positive at 1 and 2 months AGI and expressed elevated HIF1A levels (fig. S1G), together indicating that early PIN lesions are hypoxic and that HIF1A protein levels are stabilized. In addition to hypoxia, Akt signaling has been shown to induce HIF1A levels (*9*). Since Akt signaling is activated in prostatic luminal cells of Pten^(i)pe−/−^ mice [(*15*) and fig. S1H)], we determined whether it induces HIF1A in these cells, independent of hypoxia. We therefore generated organoids from Pten^L2/L2^ and Pten^(i)pe−/−^ prostates at 3 months AGI and cultured them under normoxic conditions. HIF1A protein was detected at very low levels in such organoids, despite enhanced Akt signaling activity in those from Pten^(i)pe−/−^ mice (fig. S1I). In contrast, HIF1A levels were strongly induced in both Pten^L2/L2^ and Pten^(i)pe−/−^ organoids by the pharmacological HIF1A stabilizer DMOG (Dimethyloxallyl Glycine). Collectively, these findings demonstrate that hypoxia occurs in PINs at an early stage in their formation and leads to the stabilization of HIF1A protein.

Since HIF1A signaling was activated in prostatic premalignant lesions, we investigated whether inhibiting this pathway would affect early PINs. We thus treated Pten^(i)pe−/−^ mice at 3 months AGI with PX-478, an orally bioavailable HIF1A inhibitor (*18*, *19*) for 5 days. PX-478 reduced HIF1A levels in prostates of Pten^(i)pe−/−^ mice (fig. S1J), in line with a previous study in human PCa cells (*20*). Remarkably, this treatment lowered their prostate weight by 40% (Fig. 1G), reduced PIN severity, and induced cleaved caspase 3 levels in luminal cells of PINs (Fig. 1H). Therefore, these data demonstrate that pharmacological inhibition of HIF1A in early lesions leads to the elimination of PIN cells by apoptosis.

Treatment of Pten^(i)pe−/−^ mice at 10 months AGI, i.e., the phase of PIN evolution to malignancy, with PX-478 for 1 month also reduced HIF1A levels and led to a 40% reduction in the prostate weight (fig. S1K and Fig. 1I). Regions of PINs and adenocarcinoma were detected in prostates of both vehicle- and PX-478–treated mice (Fig. 1J), but the proliferation index of epithelial cells was reduced by more than 60% with PX-478 (Fig. 1K and fig. S1K). Of note, the number of cleaved caspase 3–positive epithelial cells was low in vehicle- and PX-478–treated Pten^(i)pe−/−^ mice (fig. S1K). Moreover, a similar treatment regimen did not affect the prostate weight or histology of age-matched Pten^L2/L2^ mice (Fig. 1, I and J), demonstrating that the inhibitor does not affect nonneoplastic epithelial cells.

Pathway analysis of the genes up-regulated in the nonepithelial cell clusters of prostates of Pten^(i)pe−/−^ mice compared to Pten^L2/L2^ ones demonstrated that HIF1 signaling is also induced in microenvironmental cells, e.g., fibroblasts and leukocytes, the latter including MDSCs (tables S4 and S5). Quantification of stromal cells in H&E (hemtoxylin and eosin)-stained prostatic sections revealed that PX-478 treatment at 10 months AGI attenuates the stromal reaction in Pten^(i)pe−/−^ mice (fig. S1L). Moreover, flow cytometry analyses revealed that PX-478 reduces the proportion of total leukocytes and MDSCs in prostates of Pten^(i)pe−/−^ mice (fig. S1M). Therefore, PX-478 affects both epithelial and microenvironmental cells, together leading to a reduction in the prostate weight of Pten^(i)pe−/−^ mice at this disease stage. Collectively, our analyses indicate that HIF1A promotes cell survival in early prostatic lesions and progression to malignant tumors.

### PIN formation is independent of luminal HIF1A

To further study the role of HIF1A in prostatic luminal cells during PIN formation and evolution, we generated Pten/Hif1a^(i)pe−/−^ mice by tamoxifen treatment of PSA-CreER^T2^ mice bearing floxed Pten and Hif1a alleles. Immunohistochemical analyses demonstrated that most epithelial cells of Pten/Hif1a^(i)pe−/−^ prostates did not express HIF1A and that ENO1 (Enolase 1) expression was strongly down-regulated (fig. S2A), demonstrating that *Hif1a* was efficiently ablated in prostatic luminal cells of Pten/Hif1a^(i)pe−/−^ mice.

**Fig. 2.**
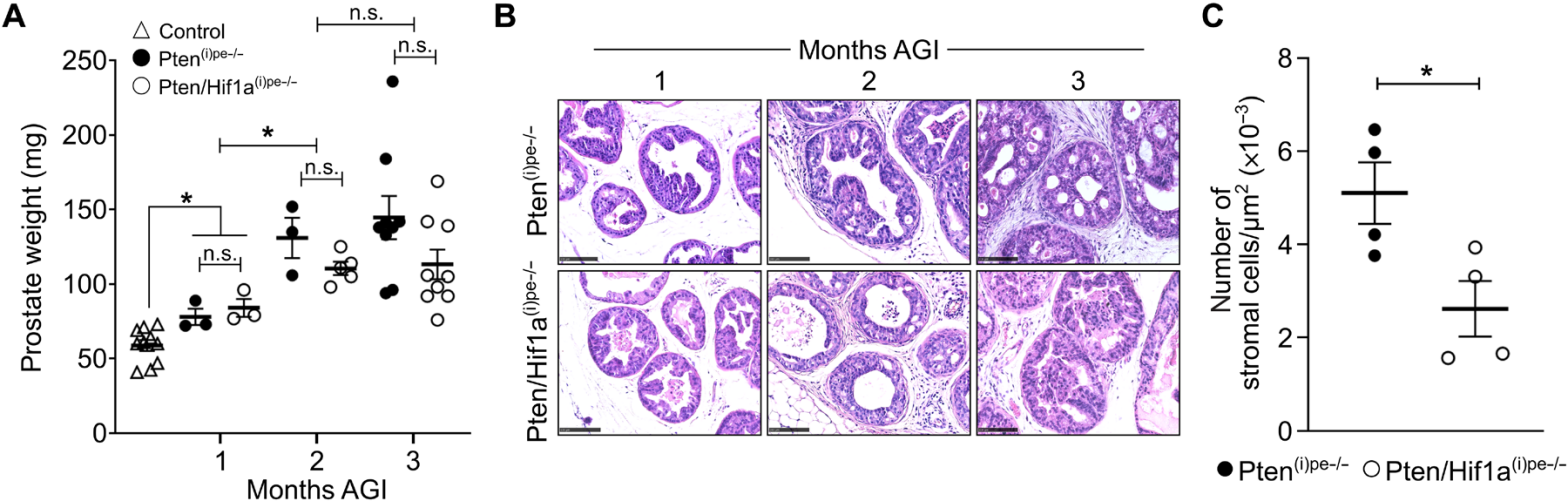
Characterization of Pten/Hif1a^(i)pe−/−^ mice. (**A**) Prostate weights of control, Pten^(i)pe−/−^, and Pten/Hif1a^(i)pe−/−^ mice at 1, 2, and 3 months AGI. *N* = 3 to 10 mice per genotype. (**B**) Representative hematoxylin and eosin (H&E) staining of the DLP of Pten^(i)pe−/−^ and Pten/Hif1a^(i)pe−/−^ mice at 1, 2, and 3 months AGI. *N* = 3 to 5 mice per condition. (**C**) Quantification of stromal cells in DLP of Pten^(i)pe−/−^ and Pten/Hif1a^(i)pe−/−^ mice at 3 months AGI. N = 4 mice per condition. Comparison between two groups was performed using a two-tailed *t* test. n.s., *P* ≥ 0.05; *P < 0.05.

The prostate weight of Pten^(i)pe−/−^ and Pten/Hif1a^(i)pe−/−^ mice was similarly induced at 1 month AGI, compared to that of wild-type mice, and was further induced between 1 and 2 months AGI. At 3 months AGI, it was however lower in Pten/Hif1a^(i)pe−/−^ mice than in Pten^(i)pe−/−^ ones, although the reduction did not reach statistical significance (Fig. 2A).

Histological analyses revealed the presence of PINs in prostates of Pten^(i)pe−/−^ and Pten/Hif1a^(i)pe−/−^ mice at 1 month AGI, which became more severe in both mouse lines at 3 months AGI (Fig. 2B). However, the time-dependent emergence of the stromal reaction in Pten^(i)pe−/−^ prostates was largely attenuated in Pten/Hif1a^(i)pe−/−^ ones (Fig. 2, B and C), indicating that luminal HIF1A does not play a major role in PIN formation but may have an effect on microenvironmental cells. Of note, early-stage PINs of Pten/Hif1a^(i)pe−/−^ mice were hypoxic (fig. S2B), as observed in those of Pten^(i)pe−/−^ ones (fig. S1G and Fig. 1F).

### HIF1A promotes glucose metabolism in luminal-C cells

**Fig. 3.**
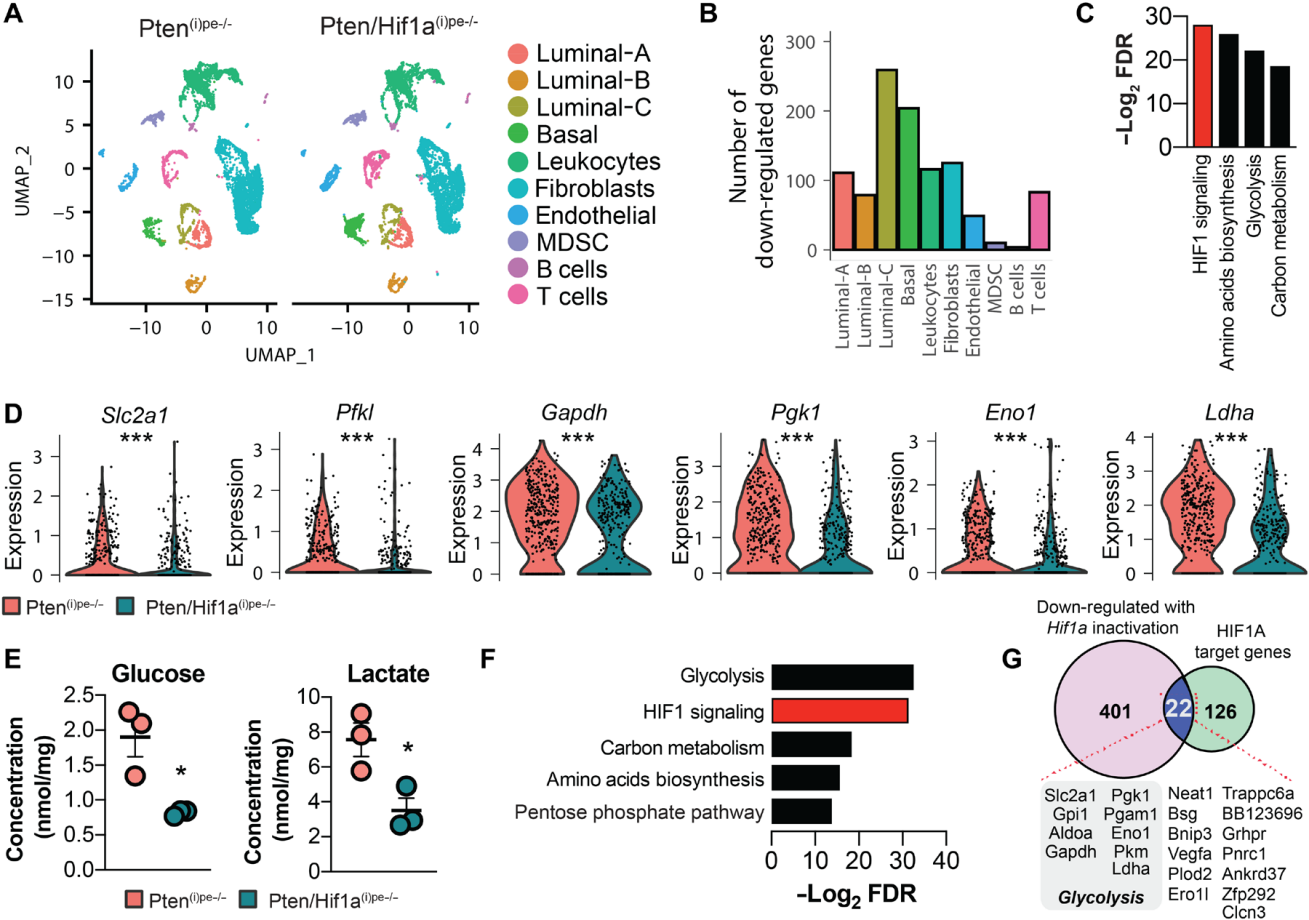
Stimulation of glucose metabolism by HIF1A in luminal-C cells. (**A**) UMAP of cells of Pten^(i)pe−/−^ and Pten/Hif1a^(i)pe−/−^ prostates at 3 months AGI. (**B**) Bar chart depicting the number of genes down-regulated in the indicated cell clusters after Hif1a inactivation. Adjusted *P* value of <0.05. (**C**) KEGG pathways analysis of the genes down-regulated in luminal-C cells of Pten/Hif1a^(i)pe−/−^ mice compared to Pten^(i)pe−/−^ ones. (**D**) Violin plots depicting transcript levels of HIF1 signaling–related genes in luminal-C cells of Pten^(i)pe−/−^ and Pten/Hif1a^(i)pe−/−^ mice. ***P < 0.001 calculated using the Wilcoxon rank sum test. (**E**) Glucose and lactate levels in Pten^(i)pe−/−^ and Pten/Hif1a^(i)pe−/−^ prostates at 3 months AGI determined by NMR. *N* = 3 mice per condition. Comparison between the two groups was performed using a two-tailed *t* test. *P < 0.05. (**F**) KEGG pathway analysis of HIF1A target genes identified by ChIP-seq of DMOG-treated Myc-CaP cells. (**G**) Venn diagram demonstrating the overlap between genes down-regulated in luminal-C cells of Pten/Hif1a^(i)pe−/−^ mice compared to Pten^(i)pe−/−^ (*P* < 0.05) and HIF1A target genes identified in Myc-CaP cells.

To characterize the impact of luminal *Hif1a* loss on the various cell types in precancerous lesions, we performed scRNA-seq on 8295 cells of dissociated Pten/Hif1a^(i)pe−/−^ mice prostates at 3 months AGI (Fig. 3A). Based on the analysis of these cells together with the age-matched Pten^(i)pe−/−^ ones described above, we identified leukocytic and nonleukocytic mesenchymal clusters, as well as epithelial cells, including luminal-C cells (Fig. 3A and table S6). Flow cytometry analysis revealed that the proportions of luminal-C cells were similar in prostates of Pten^(i)pe−/−^ and Pten/Hif1a^(i)pe−/−^ mice at 3 months AGI (fig. S3A). Moreover, immunohistochemical analysis showed strong nuclear AR staining in TROP2-positive luminal cells of Pten/Hif1a^(i)pe−/−^ mice (fig. S3B), as seen in Pten^(i)pe−/−^ ones (fig. S1E). However, differential expression analysis demonstrated that luminal-C cells contained the largest number of genes down-regulated by *Hif1a* inactivation (Fig. 3B and table S7). KEGG pathway analysis of these genes identified HIF1 signaling, biosynthesis of amino acids, glycolysis and carbon metabolism (Fig. 3C and table S8). The transcript levels of numerous glycolytic enzymes including 6-phosphofructokinase (*Pfkl*), glyceraldehyde-3-phosphate dehydrogenase (*Gapdh*), phosphoglycerate kinase 1 (*Pgk1*), enolase 1 (*Eno1*), and lactate dehydrogenase A (*Ldha*), as well as those of glucose transporter 1 (*Slc2a1*), were expressed at lower levels in luminal-C cells of Pten/Hif1a^(i)pe−/−^ mice than of Pten^(i)pe−/−^ ones (Fig. 3D). Moreover, nuclear magnetic resonance (NMR)–based metabolomics performed at 3 months AGI revealed that glucose and lactate levels were around twofold lower in Pten/Hif1a^(i)pe−/−^ prostates compared to Pten^(i)pe−/−^ ones (Fig. 3E and table S9). Thus, these results demonstrate that HIF1A promotes glucose metabolism in prostatic luminal-C cells.

We next investigated whether HIF1A directly regulates the expression of these glucose metabolism–related genes. As publicly available datasets of HIF1A DNA binding sites in prostate tumor cells were scarce, with these analyses performed on human PC-3 cells (*21*), which are of metastatic origin, and data on murine prostate cells were unavailable (*22*), we induced HIF1A protein levels in Myc-CaP cells by a 6-hour treatment with DMOG (fig. S3, C and D) and sequenced chromatin immunoprecipitated with an HIF1A antibody (ChIP-seq). HIF1A was found to be enriched in regulatory regions of around 150 genes (table S10 and fig. S3E), and de novo motif analysis identified the canonical hypoxia response element (A/G-CGTG) in 110 binding sites (fig. S3F). KEGG pathway analysis of all identified genes demonstrated that the enriched pathways were mainly metabolic networks (Fig. 3F). To identify HIF1A target genes whose expression in luminal-C cells is affected by *Hif1a* inactivation, we intersected the genes down-regulated in Pten/Hif1a^(i)pe−/−^ luminal-C cells with those identified by the ChIP-seq analysis. Among the 22 genes identified, 9 were glycolysis-related (*Slc2a1*, *Gpi1*, *Aldoa*, *Gapdh*, *Pgk1*, *Pgam1*, *Eno1*, *Pkm*, and *Ldha*) (Fig. 3G). Therefore, these results provide evidence that HIF1A directly induces the expression of various glucose metabolism–related genes in luminal-C cells of PINs.

A large number of genes were also differentially expressed in basal cells between Pten^(i)pe−/−^ and Pten/Hif1a^(i)pe−/−^ mice (Fig. 3B), demonstrating that Hif1a inactivation in luminal cells affects basal cells. Of note, pathway analysis of these genes did not identify HIF1 signaling or glucose metabolism (tables S7 and S8), in line with lack of HIF1A signaling activation in basal cells in PINs of Pten^(i)pe−/−^ mice (tables S4 and S5).

### HIF1A loss reprograms the senescence-associated secretory phenotype and promotes immune surveillance and apoptosis

**Fig. 4.**
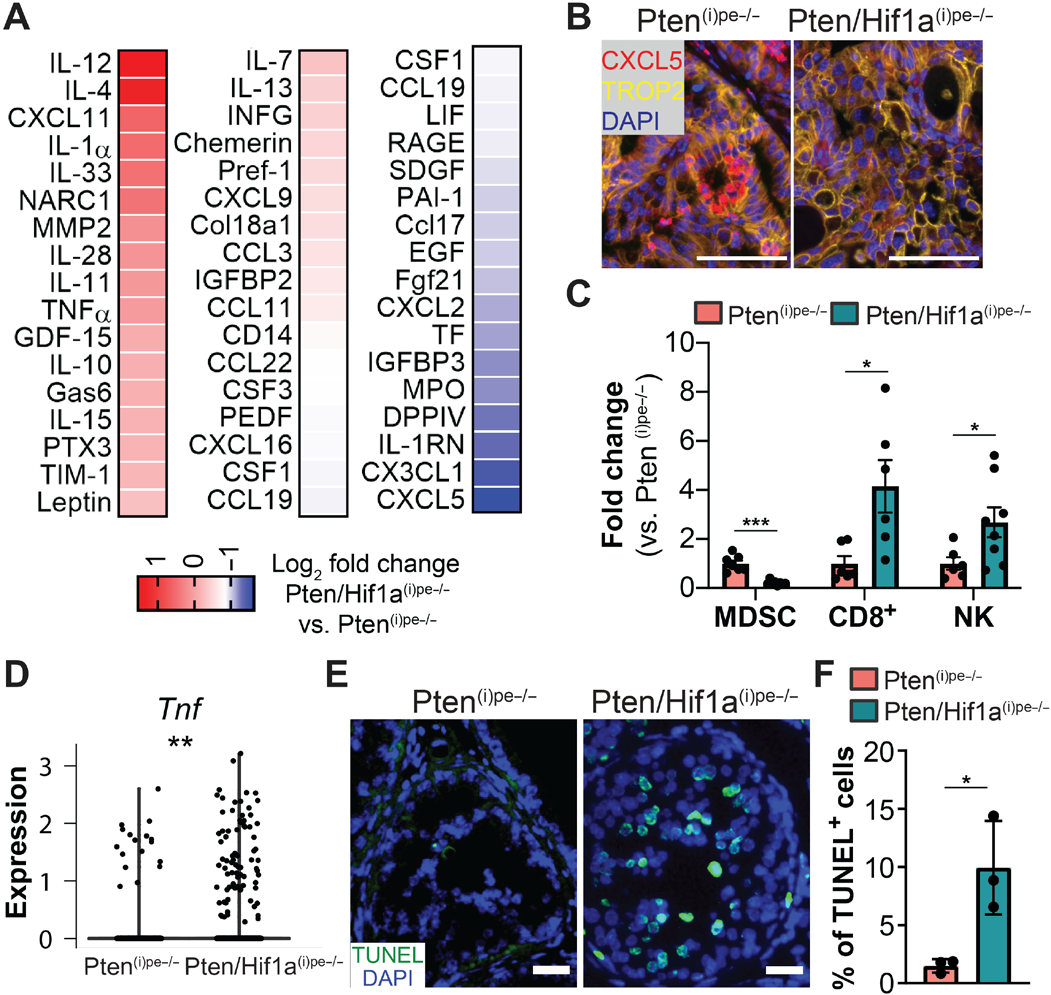
Regulation of immune surveillance by luminal HIF1A. (**A**) Cytokine profiling in FACS-sorted Pten^(i)pe−/−^ and Pten/Hif1a^(i)pe−/−^ luminal-C cells at 3 months AGI. Lysates were obtained from a pool of three prostates per condition. (**B**) Representative immunostaining of CXCL5 (red) and TROP2 (yellow) in PINs in the DLP of Pten^(i)pe−/−^ and Pten/Hif1a^(i)pe−/−^ mice at 3 months AGI. Scale bars, 50 μm. *N* = 3 mice per condition. (**C**) Flow cytometric analysis of MDSCs, CD8 + T cells, and NK cells in Pten^(i)pe−/−^ and Pten/Hif1a^(i)pe−/−^ prostates at 3 months AGI. *N* = 6 to 8 mice per condition. Comparisons between groups were performed using a two-tailed *t* test. *P < 0.05 and ***P < 0.001. (**D**) Violin plot depicting transcript levels of *Tnf* in the T cell clusters of Pten^(i)pe−/−^ and Pten/Hif1a^(i)pe−/−^ mice at 3 months AGI. **P < 0.01 calculated using the Wilcoxon rank sum test. (**E**) TUNEL assay performed on prostatic sections of Pten^(i)pe−/−^ and Pten/Hif1a^(i)pe−/−^ mice at 3 months AGI. Representative images of the DLP are shown. Scale bars, 50 μm. (**F**) Quantification of TUNEL-positive epithelial cells in the DLP of Pten^(i)pe−/−^ and Pten/Hif1a^(i)pe−/−^ mice at 3 months AGI. *N* = 3 mice per condition. Statistical significance was calculated using a two-tailed *t* test. *P < 0.05.

As Pten^(i)pe−/−^ PINs exhibit characteristics of cellular senescence at 3 months AGI (*16*), we determined the influence of HIF1A on senescence entry. The proliferation index of prostatic epithelial cells was low (<5%) in both Pten^(i)pe−/−^ and Pten/Hif1a^(i)pe−/−^ mice at 3 months AGI, and PINs were senescence-associated β-galactosidase (SA-β-gal) positive (fig. S4, A and B). However, profiling the expression of more than 40 cytokines in fluorescence-activated cell sorting (FACS)–sorted luminal-C cells of Pten^(i)pe−/−^ and Pten/Hif1a^(i)pe−/−^ prostates revealed that the levels of most investigated cytokines were altered by *Hif1a* inactivation. CXCL5 and CX3CL1, known senescence-associated secretory phenotype (SASP) components and MDSC recruiting factors (*23*–*25*), were the most down-regulated cytokines by *Hif1a* inactivation (Fig. 4A). Moreover, immunostaining studies demonstrated that CXCL5 is expressed at much lower levels in TROP2-positive cells of Pten/Hif1a^(i)pe−/−^ mice than of Pten^(i)pe−/−^ ones (Fig. 4B). In addition, flow cytometry analyses revealed an 80% reduction in the proportion of MDSCs in Pten/Hif1a^(i)pe−/−^ prostates compared to Pten^(i)pe−/−^ ones (Fig. 4C).

The cytokine screen also identified interleukin-12, a known recruiter of cytotoxic lymphocytes (*26*), as the most induced cytokine in Pten/Hif1a^(i)pe−/−^ luminal-C cells compared to Pten^(i)pe−/−^ ones (Fig. 4A). In line with this, flow cytometry analyses revealed that the proportions of cytotoxic T (CD3^+^CD8^+^) and natural killer (NK; CD3^−^CD49b^+^NK1.1^+^) cells were 4- and 2.5-fold higher in prostates of Pten/Hif1a^(i)pe−/−^ mice than of Pten^(i)pe−/−^ ones at 3 months AGI, respectively (Fig. 4C). Furthermore, differential expression analyses on the T cell cluster comprising *Cd8a*-expressing T cells, NK-T cells and NK cells, revealed that the proapoptotic cytokine *Tnf* (tumor necrosis factor) was expressed at higher levels after *Hif1a* ablation (Fig. 4D). Moreover, the number of TUNEL (terminal deoxynucleotidyl transferase–mediated deoxyuridine triphosphate nick end labeling)–positive luminal cells at 3 months AGI was more than sixfold higher in prostates of Pten/Hif1a^(i)pe−/−^ mice than of Pten^(i)pe−/−^ ones (Fig. 4, E and F). Therefore, combined inactivation of *Pten* and *Hif1a* in luminal cells does not prevent senescence entry but reprograms the SASP, leading to a reduction in the prostatic infiltration of immunosuppressive MDSCs and an increase in cytotoxic lymphocytes, together leading to apoptosis of some PIN cells.

### HIF1A promotes the plasticity and progression of PINs

**Fig. 5.**
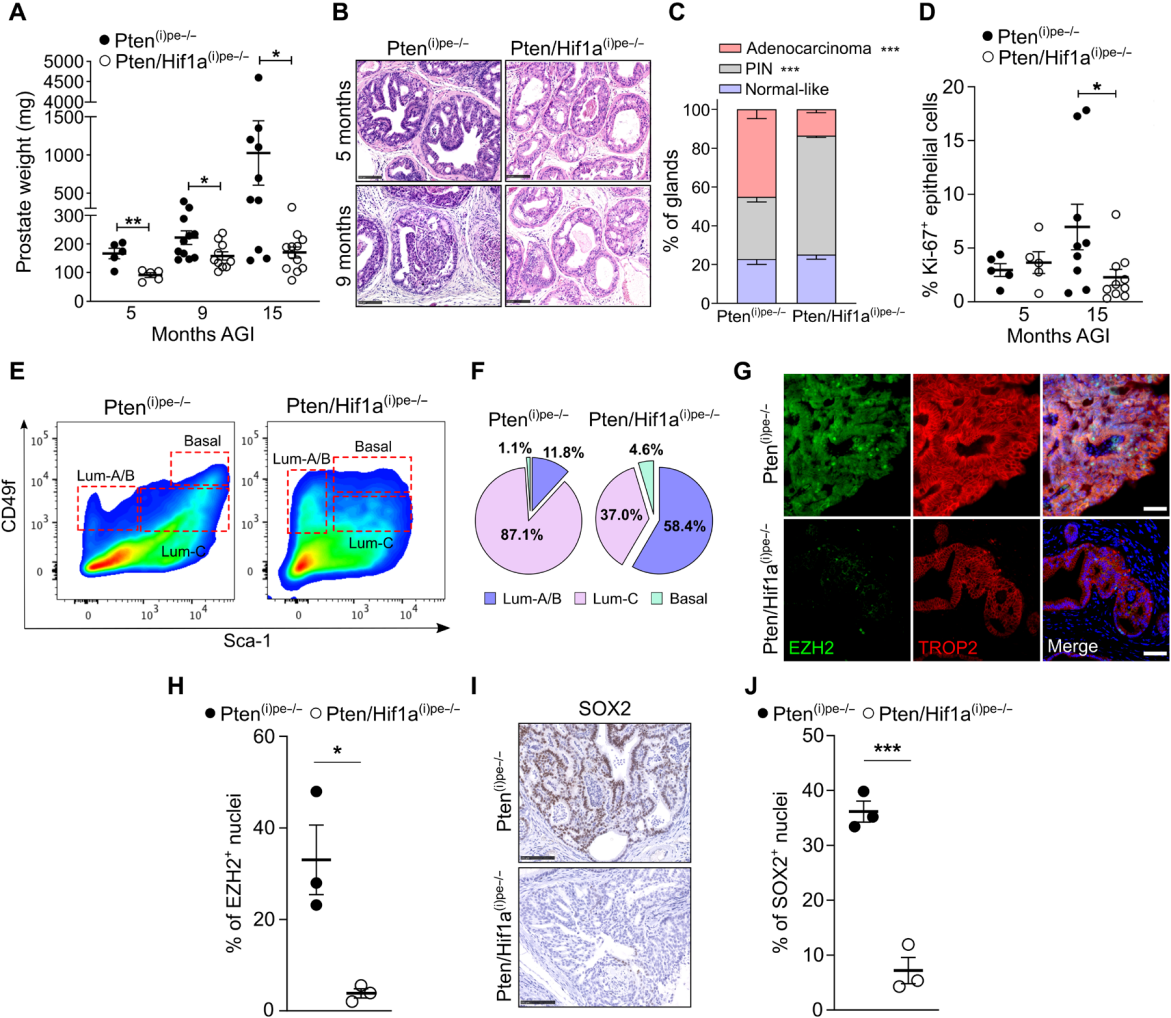
Control of PIN progression and plasticity by HIF1A. (**A**) Prostate weight of Pten^(i)pe−/−^ and Pten/Hif1a^(i)pe−/−^ mice at 5, 9, and 15 months AGI. *N* = 5 to 12 mice per condition. (**B**) H&E-stained sections of Pten^(i)pe−/−^ and Pten/Hif1a^(i)pe−/−^ prostates at 5 and 9 months AGI. Representative images of the DLP are shown. *N* = 3 to 5 mice per condition. Scale bars, 100 μm. (**C**) Quantification of gland architecture in DLP of Pten^(i)pe−/−^ (*n* = 4) and Pten/Hif1a^(i)pe−/−^ (*n* = 5) mice at 15 months AGI. (**D**) Proliferation index (Ki-67^+^ epithelial cells) in the DLP of Pten^(i)pe−/−^ and Pten/Hif1a^(i)pe−/−^ mice at 5 and 15 months AGI. *N* = 5 to 10 mice per condition. (**E**) Pseudocolor FACS plot of epithelial subsets in Pten^(i)pe−/−^ and Pten/Hif1a^(i)pe−/−^ prostates at 15 months AGI. (**F**) Pie charts depicting the proportions of epithelial subsets in Pten^(i)pe−/−^ and Pten/Hif1a^(i)pe−/−^ prostates at 15 months AGI. *N* = 3 mice per condition. (**G**) Representative immunostaining of EZH2 (green) and TROP2 (red) in the DLP of Pten^(i)pe−/−^ and Pten/Hif1a^(i)pe−/−^ mice at 15 months AGI. DAPI (blue); scale bars, 50 μm. (**H**) Quantification of EZH2-positive nuclei in luminal cells of the DLP of Pten^(i)pe−/−^ and Pten/Hif1a^(i)pe−/−^ mice. *N* = 3 mice per condition. Representative immunohistochemical detection of SOX2 (**I**), and quantification of SOX2-positive nuclei in epithelial cells of the DLP of Pten^(i)pe−/−^ and Pten/Hif1a^(i)pe−/−^ mice at 15 months AGI (**J**). Scale bars, 100 μm. *N* = 3 mice per condition. *P < 0.05; **P < 0.01; ***P < 0.001, calculated using a two-tailed *t* test.

To investigate the impact of HIF1A loss on PIN evolution, we determined the prostate weight of Pten^(i)pe−/−^ and Pten/Hif1a^(i)pe−/−^ mice up to 15 months AGI. At 5 and 9 months AGI, the prostate weight of Pten/Hif1a^(i)pe−/−^ mice was 1.8- and 1.4-fold lower than that of Pten^(i)pe−/−^ ones, respectively (Fig. 5A). Furthermore, while the prostate weight of Pten^(i)pe−/−^ mice increased by fivefold between 9 and 15 months, that of Pten/Hif1a^(i)pe−/−^ did not increase (Fig. 5A). Histologically, *Hif1a* ablation reduced the PIN severity and the stromal reaction in mice at 5 and 9 months AGI (Fig. 5B). At 15 months AGI, around 50% of the glands in the dorsolateral prostate (DLP) of Pten^(i)pe−/−^ mice contained adenocarcinoma, whereas most glands in Pten/Hif1a^(i)pe−/−^ ones contained PINs (Fig. 5C and fig. S5A). Moreover, while the proliferation index of prostatic epithelial cells of Pten^(i)pe−/−^ mice increased from an average of <5 to 8% between 5 and 15 months AGI, and even reached >15% in some prostates, it remained <5% in Pten/Hif1a^(i)pe−/−^ ones (Fig. 5D and fig. S5B). Flow cytometry analyses at 15 months AGI revealed that luminal-C and -A/B cells were the predominant epithelial subsets in Pten^(i)pe−/−^ and Pten/Hif1a^(i)pe−/−^ prostates, respectively (Fig. 5, E and F). As the luminal-C population was the major subset in both mouse lines at 3 months AGI (fig. S3A), our results show that HIF1A affects the epithelial cell state during tumor progression.

As PIN evolution was impaired by Hif1a inactivation, we compared the expression of SOX2 (SRY-box transcription factor 2) and EZH2 (Enhancer of zeste homolog 2), two known regulators of PCa progression and plasticity (*27*, *28*), in prostates of Pten^(i)pe−/−^ and Pten/Hif1a^(i)pe−/−^ mice. At 15 months AGI, both SOX2 and EZH2 were expressed at much lower levels in prostates of the latter (Fig. 5, G to J). In addition, knocking down *HIF1A* in human and mouse PCa cell lines cultured under normoxic conditions reduced the levels of SOX2 and EZH2 (fig. S5, C and D). As prostatic lesions of both Pten^(i)pe−/−^ and Pten/Hif1a^(i)pe−/−^ mice at 15 months AGI were hypoxic (fig. S5E), these data demonstrate that HIF1A promotes the expression of EZH2 and SOX2 under both hypoxic and normoxic conditions and enhances the progression of prostatic tumors.

### HIF1A signaling correlates with TGM2 expression, which is associated with disease progression

**Fig. 6.**
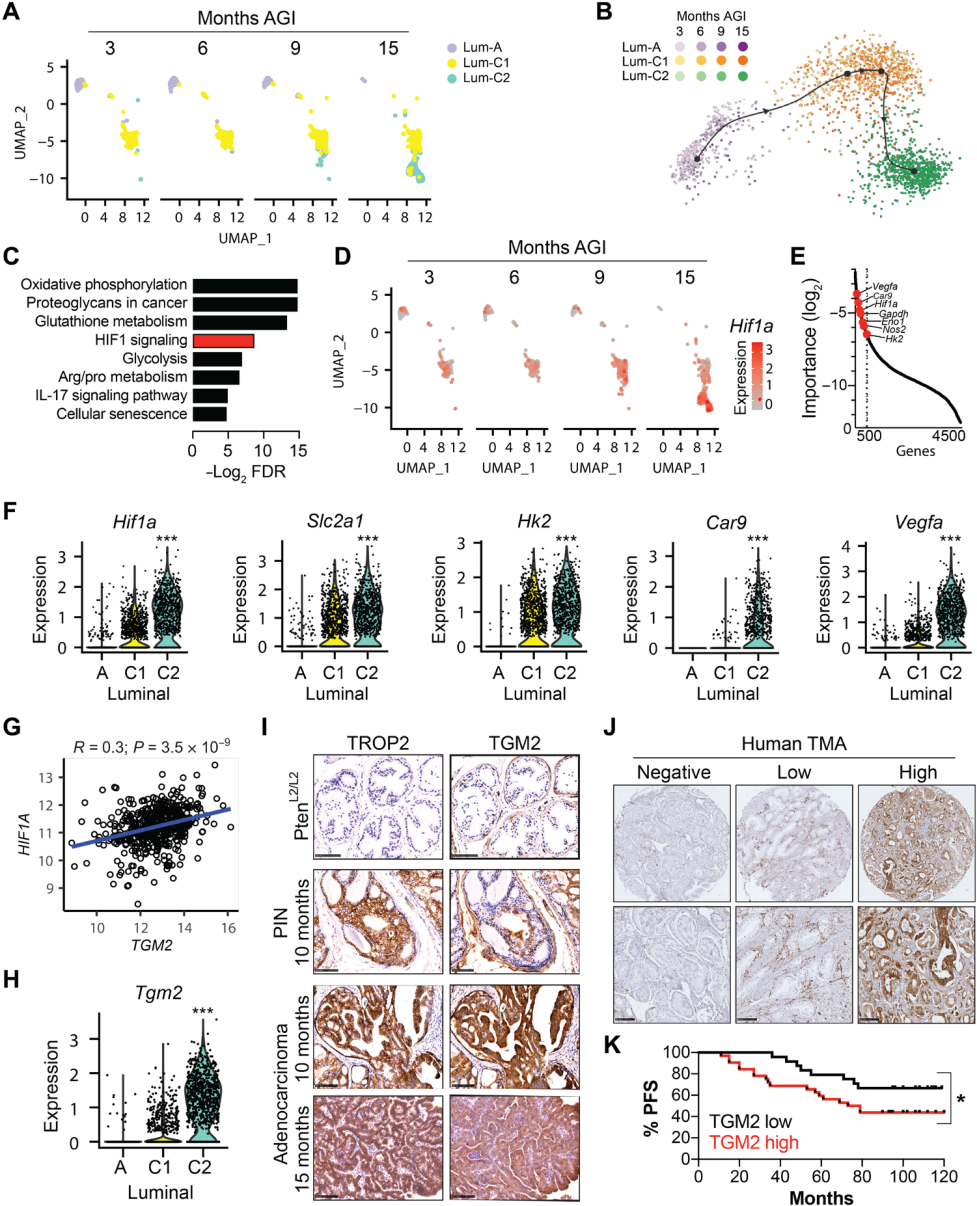
Identification of TGM2-expressing luminal cells in mouse and human PCa and association with HIF1A. (**A**) UMAP of luminal clusters in prostates of Pten^(i)pe−/−^ mice at 3, 6, 9, and 15 months AGI. (**B**) Cellular trajectory colored by luminal subset. Color gradients distinguish cells at the various time points AGI. (**C**) KEGG pathway analysis of the top 500 genes in the trajectory inference analysis. (**D**) Hif1a transcript levels in the various luminal subsets at the indicated time points AGI. (**E**) Plot of gene importance depicting HIF1A signaling–related genes among the top 500 genes (dashed vertical line). (**F**) Violin plots depicting the transcript levels of Hif1a and its target genes in luminal-A, -C1, and -C2 cells. Data were obtained from samples at 3, 6, 9, and 15 months AGI. ***P < 0.001 calculated using the Wilcoxon rank sum test. (**G**) Analysis of the correlation between HIF1A and TGM2 transcript levels in human PCa transcriptomes on the TCGA. Pearson’s correlation coefficient was used to determine the association between the two genes. (**H**) Violin plot depicting the transcript levels of Tgm2 in luminal-A, -C1, and -C2 cells. (**I**) Immunohistochemical staining of TROP2 and TGM2 in prostatic sections of Pten^L2/L2^ and Pten^(i)pe−/−^ mice at 10 and 15 months AGI. Representative images of the DLP are shown. *N* = 3 mice per condition. Scale bars, 100 μm. (**J**) Representative immunohistochemical staining of TGM2 in human prostate adenocarcinoma in TMA sections (*n* = 56 patients). Scale bars, 100 μm. (**K**) Kaplan-Meier plots demonstrating the PFS of patients with high prostatic TGM2 expression (*n* = 32 patients) and those with low levels of TGM2 (*n* = 24 patients). *P < 0.05, calculated using the Gehan-Breslow-Wilcoxon test.

To shed light on the mechanisms underlying the progression of luminal cells, we performed droplet-based scRNA-seq on dissociated Pten^(i)pe−/−^ prostates at 6 and 15 months AGI, corresponding to intermediate-stage PINs and malignant tumors, respectively, and combined these datasets with those obtained from early- (3 months AGI; see above) and late-stage [9 months AGI (*17*)] PIN lesions. These analyses show a time-dependent reduction in the proportion of luminal-A cells between 3 and 15 months AGI and a concomitant increase in that of the luminal-C subset (Fig. 6A). A second luminal-C cluster (termed luminal-C2), which was previously identified in Pten^(i)pe−/−^ prostates at 9 months AGI (*17*), represented around 90% of luminal epithelial cells at 15 months AGI (Fig. 6A and table S11). These cells express *Krt4* and *Tacstd2*, but lower levels of the AR target genes *Pbsn* and *Nkx3-1* than the other luminal C cluster (termed hereafter luminal-C1) (fig. S6A). Hence, together, these findings demonstrate that distinct luminal cell states are associated with different disease stages, where luminal-C1 and -C2 cells are the predominant luminal subsets in PINs and malignant tumors, respectively.

Trajectory inference analysis on these clusters identified three major nodes connected by a linear trajectory (Fig. 6B). The first node mainly consisted of cells isolated at 3 and 6 months AGI, all of which were luminal-A cells (Fig. 6B). The second node was enriched in cells isolated at 9 months AGI and contained mostly luminal-C1 cells, whereas the third node contained luminal-C2 cells almost exclusively obtained at 15 months AGI (Fig. 6B). Enrichment analysis of the top 500 features, based on gene importance implicated in the cells’ trajectory, identified cellular senescence and a number of metabolic pathways, including those involved in glucose, glutathione, and amino acid metabolism (Fig. 6C). HIF1 signaling was among the identified pathways, with *Hif1a* and a number of its target genes including *Vegfa*, *Car9*, and *Eno1* identified (Fig. 6, C to E). Furthermore, transcript levels of *Hif1a* and a number of its target genes including *Slc2a1*, *Hk2*, *Car9*, and *Vegfa* were higher in luminal-C1 cells than in luminal-A cells and were further increased in luminal-C2 cells (Fig. 6F). These results demonstrate that HIF1A signaling, which is already induced in early hypoxic PIN lesions compared to normal glands (Fig. 1, C to F), is further enhanced during disease progression and is associated with a switch in cell state from luminal-C1 to -C2.

Differential expression analysis revealed that the transcript levels of almost 400 genes were induced in luminal-C2 cells compared to luminal-A and -C1 ones (table S12). ONECUT2 (one cut domain, family member 2), which has been recently linked to cellular plasticity in PCa (*21*), was the most up-regulated gene in luminal-C2 cells compared to the other luminal subsets (table S12). However, HIF1A knockdown in PCa cells did not affect ONECUT2 levels (fig. S6B).

To identify genes in the luminal-C2 signature that are associated with HIF1A-driven PIN progression, we overlapped the top 100 DEGs in luminal-C2 cells with those identified in the trajectory inference analysis and correlated the expression of the common genes with those of HIF1A in human PCa transcriptomes on The Cancer Genome Atlas (TCGA) database (fig. S6C). Among the 55 genes found in common, the transcript levels of four directly correlated with those of HIF1A (fig. S6C). As the top 2 genes, *Itga2* (Cd49b) and *Vcam1*, are highly expressed in nonepithelial cells, namely, immune and endothelial cells (*29*, *30*), they were not further investigated. The next identified gene was *Tgm2* (fig. S6C, Fig. 6G, and table S12), which encodes an enzyme that has multiple cellular functions and is known to play both pro- and antitumoral roles (*31*). Very low *Tgm2* transcript levels were present in luminal-A cells, whereas higher levels were detected in luminal-C1 cells and the highest levels in luminal-C2 cells (Fig. 6H), following a similar expression pattern to *Hif1a* and its targets genes (Fig. 6F). In addition, in human PCa transcriptomes of the TCGA PRAD (Prostate Adenocarcinoma) cohort, *TGM2* levels positively correlated with an HIF1A signature consisting of 10 known HIF1A targets (*SLC2A1*, *HK2*, *PFKA*, *ALDOA*, *GAPDH*, *ENO1*, *LDHA*, *CA9*, *BNIP3*, and *VEGFA*), a human PCa-specific hypoxia signature (*32*), and a general hypoxia signature obtained from the molecular signatures database (*33*) (fig. S6D), together demonstrating the association of TGM2 expression with HIF1A/hypoxia signaling.

Immunohistochemical analyses demonstrated that TGM2 is not expressed in luminal cells of wild-type prostates (Fig. 6I). At 10 and 15 months AGI, TROP2-positive adenocarcinoma regions of Pten^(i)pe−/−^ mice were TGM2 positive, whereas in PINs, only a subset of TROP2-positive cells expressed TGM2 (Fig. 6I). These results show that TGM2 levels in TROP2-expressing luminal-C cells are associated with malignancy. Moreover, immunoblotting revealed that TGM2 expression was lower in FACS-sorted luminal-C cells of Pten/Hif1a^(i)pe−/−^ mice compared to those of Pten^(i)pe−/−^ ones (fig. S6E). In addition, immunohistochemical analyses on prostatic sections demonstrated that TROP2-positive luminal cells of Pten/Hif1a^(i)pe−/−^ mice expressed markedly lower levels of TGM2 compared to their Pten^(i)pe−/−^ counterparts (fig. S6F). In line with these data, genetic and pharmacological inhibition of HIF1A in PCa cells led to reduced levels of TGM2 (fig. S6G). Collectively, these data demonstrate that HIF1A plays a key role in regulating TGM2 expression and the emergence of TGM2-expressing luminal-C cells.

In view of these findings, we determined whether TGM2 levels are predictive for disease progression in humans. Analysis of the TCGA PRAD cohort revealed that TGM2 expression was higher in prostatic tumors with a Gleason grade of 9 compared to those with a grade of 6, as well as to normal prostates (fig. S6H). In addition, we analyzed the expression of TGM2 in prostatic tumoral sections of 56 treatment-naïve patients with PCa who had undergone radical prostatectomy (Fig. 6J) and found that patients expressing high levels of TGM2 had lower progression-free survival (PFS) (Fig. 6K), with the average PFS shortened by 17 months compared to those expressing low TGM2 levels (table S13). The baseline characteristics of both groups of patients, including PSA levels and Gleason scores, were similar (table S13). As both TGM2-high and -low/negative groups comprised patients classified as intermediate risk (table S13), TGM2 may be used as a marker for the early identification of patients with higher risk of disease recurrence. Collectively, these findings demonstrate that TGM2 expression correlates with HIF1A signaling in mice and human prostatic tumors, has potential prognostic value in predicting relapse in patients with PCa, and may thus be used to guide their clinical management.

## DISCUSSION

Advances made in the management of PCa have markedly improved survival rates in men with localized disease, but current treatments have considerable side effects (*2*). Moreover, no reliable biomarkers are available to predict relapses, and patients with metastatic disease have limited treatment options and an overall poor prognosis. Therefore, preventing the progression of precancerous lesions to malignant tumors would circumvent these outcomes and is an attractive strategy to manage PCa. To unravel novel mediators of PIN evolution, we analyzed Pten^(i)pe−/−^ mice, which develop PINs that evolve to adenocarcinoma and thus closely recapitulate the natural history of the disease in humans. We demonstrate that hypoxic signaling is activated in early PINs, a phenomenon previously reported in malignant tumors (*11*, *12*), and identify HIF1A as a major driver of PIN progression.

Our study shows that the protumorigenic roles of HIF1A are both tumor cell intrinsic and extrinsic. In line with the known role of HIF1A in regulating glucose metabolizing pathways (*34*), our single-cell and ChIP-seq analyses demonstrate that HIF1A induces the expression of a number of glycolytic enzymes in luminal-C cells, the major epithelial subpopulation in PINs. Moreover, we found that luminal HIF1A promotes an immunosuppressive SASP, characterized by elevated levels of MDSC-recruiting factors, including CXCL5 and CX3CL1 (*23*). Both chemokines have been previously shown to promote the progression of PCa (*35*, *36*), and Wang *et al.* (*37*) demonstrated that the CXCL5-CXCR2 axis plays an important role in the recruitment of MDSCs to prostatic tumors and that targeting it impairs disease progression. Furthermore, CXCL5 has been shown to be induced in prostates of castrated PTEN^pc−/−^ mice in which PTEN is deleted in the prostate by a probasin-driven Cre recombinase and to promote MDSC infiltration (*38*). In contrast, CX3CL1 was down-regulated in castrated Pten^PC−/−^ tumors compared to sham-operated ones. Thus, mechanisms underlying MDSC recruitment by tumoral HIF1A and in response to castration are only partially overlapping. Lactate secreted by cancer cells has also been shown to increase MDSC tumoral infiltration (*39*). Therefore, HIF1A-mediated induction of glycolysis and lactate production by PINs, as well as of CXCL5 and CX3CL1 levels, promotes MDSC recruitment and thus contributes to the immunologically “cold” phenotype of PCa (*40*). Inactivating *Hif1a* enhances the prostatic infiltration of cytotoxic lymphocytes in premalignant lesions and eliminates some PIN cells by apoptotic cell death.

Our data also demonstrate that HIF1A enhances the expression of SOX2 and EZH2 in prostatic lesions and in human and mouse PCa cells and favors a high plasticity state. These factors have been shown to promote lineage plasticity in PCa (*27*, *28*), but as our ChIP-seq analyses did not identify HIF1A binding sites in regulatory regions of the *Sox2* and *Ezh2* genes, their regulation by HIF1A is likely indirect.

Our scRNA-seq analyses revealed that the evolution of PINs to malignant tumors is associated with a shift in the luminal cell state initially from A to C1, followed by C1 to C2, in a process characterized by activation of HIF1A signaling. Since luminal-C2 cells express lower levels of AR target genes compared to their C1 counterparts, they may represent a luminal subset with intrinsic androgen-insensitive properties and hence resistant to androgen-deprivation therapy. Moreover, our data show that Onecut2, which has been shown to be a driver of lineage plasticity in PCa cells (*21*), is overexpressed in luminal-C2 cells compared to the other subsets, which suggests a role for this factor in promoting luminal plasticity in prostatic lesions. However, its levels are not affected by HIF1A knockdown in PCa cells, in agreement with previous work (*21*). As ONECUT2 has been shown to synergize with hypoxia/HIF1A signaling though activation of SMAD3 (SMAD family member 3) (*21*), we propose that plasticity in prostatic lesions may be driven by a cross-talk between HIF1A and ONECUT2.

We identified TGM2 as a marker of luminal-C2 cells. TGM2 has been shown to be overexpressed in a number of cancer types and is known to play tumor-promoting roles, such as induction of proliferation, invasion, and angiogenesis (*31*). Its expression has also been linked to hypoxic response and has been shown to be induced in brain metastasis compared to primary breast tumors (*41*). However, the clinical relevance of TGM2 in PCa has not been previously investigated (*42*). We show that TGM2 is induced by HIF1A in prostatic lesions, correlates with HIF1A signaling in mice and humans, and is a biomarker for early relapse in patients with PCa who had undergone radical prostatectomy. Jang *et al.* (*43*) have reported that TGM2 expression is induced by HIF1A to enable cellular survival under hypoxic conditions by inhibiting apoptosis. Moreover, TGM2 has been shown to induce HIF1A expression through activating nuclear factor kB (NF-kB) signaling (*44*). It is therefore tempting to speculate that hypoxia in PINs stabilizes HIF1A, which induces the expression of TGM2 to promote cell plasticity and the malignant evolution of these lesions. TGM2 in turn may act as a sustaining factor by further inducing HIF1A expression. Luminal-C2 cells, which overexpress TGM2, also express higher HIF1A transcript levels than the other luminal subsets. Furthermore, Han *et al.* (*45*) demonstrated that overexpression of TGM2 in human PCa LNCaP cells induces an epithelial-mesenchymal transition and a reduction in AR expression. Thus, TGM2 may contribute to PCa cell plasticity.

We also investigated the efficacy of pharmacologically targeting HIF1A signaling in PINs using the orally bioavailable HIF1A inhibitor PX-478. This molecule has been previously shown to have antitumor activities in a number of mouse models of cancer (*19*, *46*) and was well tolerated by patients with cancer in a phase 1 clinical trial (*18*). We show that treatment of Pten^(i)pe−/−^ mice at 3 and 10 months AGI with PX-478 elicits profound antitumor effects, including the induction of apoptosis in early PIN lesions, and a decrease in the proliferation of late ones, which phenocopies the effects of combined inactivation of Pten and Hif1a in luminal cells. We also show that PX-478 treatment affects multiple cell types in the microenvironment, including MDSCs, which mirrors the reduction in MDSC prostatic infiltration in Pten/Hif1a^(i)pe−/−^ mice. However, as our scRNA-seq data revealed that MDSCs in prostates of Pten^(i)pe−/−^ mice have enhanced HIF1A signaling, PX-478 may also act directly on these cells, in addition to the potential disruption of their recruitment by luminal cells.

In conclusion, we demonstrate that HIF1A plays pivotal roles in the evolution of PIN lesions, through regulating energy metabolism, the secretome, and plasticity of PIN cells, as well as remodeling the microenvironment. Therefore, inhibiting HIF1A signaling is a promising strategy for PCa prevention. Moreover, as there is no consensus on the optimal management of patients with localized PCa (*47*), TGM2, which we identified as a promising prognostic marker in humans, may be used to guide clinical decision-making.

## MATERIALS AND METHODS

### Study design

The objective of the study was to identify novel mediators driving the progression of prostatic precancerous lesions to malignant tumors. To characterize the role of HIF1A in PIN evolution, we generated cohorts of Pten/Hif1a^(i)pe−/−^ mice and analyzed them, along with age-matched Pten^(i)pe−/−^ ones, at multiple time points AGI. Investigators were not blinded to animals’ genotypes. The impact of Hif1a loss in luminal cells on prostate weight and histology, cell proliferation, and apoptotic induction was determined by three investigators.

Pten^(i)pe−/−^ mice were randomized to receive either vehicle or PX-478. Investigators were not blinded to animal treatments. However, the characterization of PX-478’s effects on prostate weight and histology, apoptosis, and epithelial cell proliferation was performed by three investigators. Detailed descriptions of the experimental methods, including the number of mice, are included in the study. No sample size calculation was performed, and no outliers were excluded. In vitro results described in the study are representative of two biological replicates obtained from independent experiments.

### Mice

The generation of Pten^(i)pe−/−^ and Pten^L2/L2^ mice was as described (*15*). PSA-Cre-ER^T2(tg/0)^/Pten^L2/L2^ mice (*15*) were intercrossed with mice in which *Hif1a* alleles are floxed (*48*), to generate PSA-Cre-ER^T2(tg/0)^/Pten^L2/L2^/Hif1a^L2/L2^ and PSA-Cre-ER^T2(0/0)^/Pten^L2/L2^/Hif1a^L2/L2^. At 8 weeks of age, these mice were treated with tamoxifen (1 mg per mouse, intraperitoneally) for five consecutive days (*15*) to generate Pten/Hif1a^(i)pe−/−^ and Pten^L2/L2^/Hif1a^L2/L2^ (control) mice.

Mice breeding and maintenance were done in the accredited IGBMC/ICS animal house (C67-2018-37), in compliance with French and EU regulations on the use of laboratory animals for research. Animal experiments were approved by the ethical committee Com’Eth (Comité d’Ethique pour l’Expérimentation Animale, Strasbourg, France) and the French ministry of Higher Education and Research (#3834-2016012817529706v5).

### PX-478 treatment

PX-478 2HCl (Selleck Chemicals, catalog no. S7612) was dissolved in water and orally administered to mice at a daily dose of 20 mg/kg. Mice were treated with vehicle or PX-478 for five consecutive days or for five consecutive days followed by 2 days without intervention for 4 weeks.

### Histological examination

Hematoxylin and eosin (H&E) staining was performed on 5-μm paraffin-embedded prostate sections according to standard protocols. Histopathological assessment was performed on the DLP, and representative images are provided.

### *SA-*β*-gal staining*

SA-β-gal staining was performed on 10-μm frozen prostate sections using the senescence β-Galactosidase Staining Kit (CST 9860), following the manufacturer’s instructions. Sections were counterstained with hematoxylin.

### TUNEL assay

Apoptosis detection in prostatic tissue sections was performed using the In Situ Cell Death Detection kit (Roche ref. 11684795910). Sections were mounted using Fluoromount-G Mounting Medium with 4′,6-diamidino-2-phenylindole (DAPI; Invitrogen, 00-4959-52).

### Immunostaining

Five-micrometer paraffin-embedded prostate sections were deparaffinized and subjected to heat-induced antigen retrieval by incubating them for 20 min in a pressure cooker with SignalStain Citrate Unmasking Solution (10×) (CST 14746). The following primary antibodies were used: HIF1A (Abcam, ab51608; dilution 1:100), CA-IX (Thermo Fisher Scientific, PA1-16592; dilution 1:1000), ENO1 (Abcam, ab155102; dilution 1:200), cleaved caspase 3 (CST, 9664; dilution 1:200), Ki-67 (Thermo Fisher Scientific, MA5-14520; dilution 1:200), Cxcl5 (Thermo Fisher Scientific, BS-2549R; dilution 1:200), TROP2 [R&D systems, AF1122; dilution 1:50 (immunofluorescence detection); Abcam, ab214488; dilution 1:200 (immunohistochemical detection)], EZH2 (CST, 5246; dilution 1:200), SOX2 (Abcam, ab92494; dilution 1:100), phospho-Akt (S473) (CST, 4060S; dilution 1:200), TGM2 (CST, 3557; dilution 1:100), and AR (Abcam, ab108341; dilution 1:200).

For immunofluorescence detection, the following secondary antibodies were used at a 1:400 dilution: goat anti-rabbit immunoglobulin G (IgG) (H + L) highly cross-adsorbed secondary antibody, Alexa Fluor Plus 488 (Invitrogen, A32731); goat anti-mouse IgG (H + L) highly cross-adsorbed secondary antibody, Alexa Fluor Plus 555 (Invitrogen, A32727); and donkey anti-goat IgG (H + L) highly cross-adsorbed secondary antibody, Alexa Fluor Plus 647 (Invitrogen, A32849). Sections were mounted using Fluoromount-G Mounting Medium with DAPI (Invitrogen, 00-4959-52).

For immunohistochemical detection, one drop of SignalStain Boost immunohistochemical detection reagent [rabbit (CST 8114) or mouse (CST 8125)] was added to each section. The SignalStain DAB Substrate Kit (CST 8059), which contains the DAB chromogen concentrate and its diluent, was used to develop the signal. The sections were counterstained with hematoxylin and mounted.

### Hypoxia determination

Mice were administered pimonidazole HCl (60 mg/kg body weight) by intraperitoneal injection following the manufacturer’s instructions (HP1-100Kit) and euthanized 90 min later. Harvested prostates were fixed overnight in 4% paraformaldehyde (PFA) and subsequently embedded in paraffin. Five-micrometer sections were prepared and deparaffinized, and antigen retrieval was performed as described above. The antipimonidazole primary antibody (HP1-100Kit) was used at a dilution of 1:50.

### Microscopic acquisition

Fluorescence images were acquired using an upright motorized microscope (Leica DM 4000 B) fitted with the CoolSNAP HQ2 (Photometrics) and the Micro-Manager software, using the objectives 20× HC PL FLUOTAR [numerical aperture (NA), 0.5] and 40× N PLAN (NA, 0.65). The Fiji software was used for image editing and for quantifying TUNEL- and EZH2-positive cells. Slides were scanned in bright-field mode using the NanoZoomer digital slide scanner (Hamamatsu) and analyzed using the NDP.view2 Viewing software (Hamamatsu).

Representative images of the DLP are provided. Quantification of Ki-67– and SOX2-positive cells as well as stromal cells in the DLP was performed using the QuPath software (*49*).

### FACS analysis

#### Tissue dissociation

Mice prostates were dissociated into single cells following a described method (*50*), with minor modifications. Briefly, prostates were mechanically minced using razor blades, and disrupted tissue pieces were incubated in Dulbecco’s modified Eagle’s medium (DMEM) [glucose (4.5 g/liter), GlutaMAX, 10% fetal calf serum (FCS), and 1% penicillin/streptomycin (P/S)] supplemented with collagenase type I (1 mg/ml; 17018029 Thermo/Gibco) for 1 hour at 37°C with shaking. Samples were then spun down and incubated with trypsin/0.05% EDTA (Invitrogen/Gibco, catalog no. 25300) for 5 min in a 37°C water bath. DMEM containing 500 U of recombinant RNase-free DNase I (Roche, 04716728001) was added to the cell suspension, and single cells were obtained by passing the suspension through a 20-gauge needle at least 10 times. A 40-μm cell strainer was used to eliminate remaining clumps, and the suspension was washed once in PBS.

#### Immunophenotyping experiments

Dissociated prostates were incubated with anti-CD16/32 antibody (BD Pharmingen; 1:50) for 15 min on ice. To quantify total leukocytes (CD45^+^) and MDSCs (CD11b^+^Gr-1^+^), cells were incubated with anti-CD45 (BioLegend; ref. 103128; conjugation: Alexa Fluor 700), anti-CD11b (eBioscience; ref. 45-0112-82; conjugation: PerCP-Cy5.5), and anti–Ly-6G/Ly-6C (Gr-1) [eBioscience; ref. 11-5931-82; conjugation: fluorescein isothiocyanate (FITC)] antibodies for 15 min on ice. All antibodies were diluted 1:200 in DMEM [glucose (4.5 g/liter), 1% P/S, and 2% bovine serum albumin, without phenol red].

To quantify CD8^+^ T cells (CD3^+^CD8^+^) and NK cells (CD3^−^CD49b^+^NK1.1^+^), samples were incubated with anti-CD3ε (BioLegend; ref. 100328; conjugation: PerCP/Cy5.5; dilution 1:50), anti-CD8a (eBioscience; ref. 56-0081-80; conjugation: Alexa Fluor 700; dilution: 1:100), anti-CD49b (eBioscience; ref. 11-5971-82; conjugation: FITC; dilution: 1:100), and anti-NK1.1 (eBioscience; ref. 25-5941-82; conjugation: PE-Cyanine7; dilution: 1:100) antibodies for 15 min on ice. Cells were analyzed using a BD LSR II flow cytometer and the FlowJo software.

#### FACS of living cells for scRNA-seq

Dissociated prostates were stained with DAPI, and living cells (DAPI negative) were FACS sorted using BD FACSAria Fusion flow cytometer.

#### Luminal subset quantification and sorting

Epithelial subsets were quantified and sorted following a previously described protocol (*17*, *51*). Briefly, dissociated prostates were stained with antibodies against CD31 (eBioscience, ref. 11-0311-85; conjugation: FITC; dilution 1:250), CD45 (eBioscience, ref. 11-0451-85; conjugation: FITC; dilution 1:250), TER-119 (eBioscience, ref. 11-5921-85; conjugation: FITC; dilution 1:250), CD49f (eBioscience, ref. 12-0495-83; conjugation: PE; dilution 1:25), and Ly-6A/E (Sca-1) (eBioscience, ref. 17-5981-82; conjugation: APC; dilution 1:75). Luminal-A/B cells were defined as Lin (CD31, CD45, and TER-119) negative, Sca-1^−^, and CD49f^+^, while luminal-C cells were defined as Lin^−^, Sca-1^+^, and CD49f^med^. Cell sorting was performed using a BD FACSAria Fusion flow cytometer, whereas cell quantifications and analysis were performed using a BD LSR II flow cytometer and the FlowJo software.

### Multiplex cytokine array

The levels of cytokines in protein lysates of FACS-sorted luminal-C cells were quantified using the Proteome Profiler Mouse XL Cytokine Array (ARY028) Kit following the manufacturer’s instructions. Chemiluminescence images were captured using Amersham Imager 600 (GE Healthcare).

### Western blotting

Protein lysates were prepared from samples using radioimmunoprecipitation assay buffer, supplemented with protease (05892970001; Sigma-Aldrich) and phosphatase PhoSTOP (PHO SS-RO; Sigma-Aldrich) inhibitors. Protein concentrations in samples were determined using Bradford assay (Abcam, ab119216). Equal amounts of proteins were resolved on SDS–polyacrylamide gel electrophoresis and transferred onto nitrocellulose membranes using Trans-blot turbo transfer system (Bio-Rad).

The following primary antibodies were used at a 1:1000 dilution: HIF1A (CST 36169), Eno1 (Abcam, ab155102), Hk2 (CST, 2867), Gapdh (CST, 2118), Pten (CST, 9559), SOX2 (CST, 3579), EZH2 (CST, 5246), Ldha (Thermo Fisher Scientific, PA5-27406), phospho-Akt (S473) (CST, 4060S), ß-actin (SCBT, SC-47778), ONECUT2 (Proteintech, 21916-1-AP), histone H3 (CST, 4499S), and vinculin (SCBT, SC-25336). Anti-mouse IgG (CST, 7076S) and anti-rabbit IgG (CST, 7074S) horseradish peroxidase–linked antibodies were used at a dilution of 1:5000. Lightning Plus-ECL Enhanced Chemiluminescence Substrate (Perkin-Elmer, ref. NEL104001EA) was used to develop the signal, which was detected using the Amersham Imager 600 (GE Healthcare).

### Organoid cultures

Prostate organoid cultures were established from Pten^L2/L2^ and Pten^(i)pe−/−^ mice at 3 months AGI following previously described protocols (*52*, *53*). Briefly, prostates were dissociated into single cells, which were embedded in Matrigel (Corning, 356231) and seeded in 24-well plates (40 μl of Matrigel drop per well). Organoid medium was prepared by adding B27 (50× diluted; Gibco, 17504044), *N*-acetyl cysteine (1.25 mM; Sigma-Aldrich, A9165-25G), Hepes (10 mM; Gibco, 15630080), GlutaMAX (100× diluted; Gibco, 35050061), and P/S [1% (v/v); Gibco, 15140122] to advanced DMEM/F-12 (Gibco, 12634010). Complete medium was prepared by adding EGF (50 ng/ml; PeproTech, 315-09), Noggin (100 ng/ml; PeproTech, 120-10C), R-spondin 1 (500 ng/ml; PeproTech, 120-38), dihydrotestosterone (1 nM; Sigma-Aldrich, A8380-1G), Y-27632 (10 μM; Tocris, 1254), and A83-01 (200 nM; PeproTech, 9094360) to the organoid medium. Upon solidification of the Matrigel domes, 500 μl of complete medium was added to each well.

### Cell culture

Mycoplasma-free LNCaP, DU-145, PC-3, C42B, and Myc-CaP cells were obtained from the American Type Culture Collection and cultured in DMEM [4.5 g/liter, 10% FCS, and 1% P/S]. Cells were maintained in a culture incubator at 37°C and 5% CO_2_.

### In vitro treatments

DMOG (Tocris, 4408) treatments were performed at a final concentration of 1 mM for the indicated time points. PX-478 (Selleck Chemicals, catalog no. S7612) treatments were performed at a final concentration of 50 μM for 24 hours.

### RNA interference

Human and mouse PCa cell lines were transiently transfected with a pool of three anti-human HIF1A small interfering RNA (siRNA) (Riboxx GmbH, Radebeul, Germany; Gene ID: 3091) or three anti-mouse Hif1a siRNA (Silencer Select siRNA, Thermo Fisher Scientific, Gene ID: 15251, siRNA ID: s67530, catalog no. 4390771), respectively, or a negative control (Silencer Select Negative Control No. 1 siRNA, catalog no. 4390843). Briefly, 50 pmole of siRNA or negative control was mixed with 1.5 μl of Lipofectamine 2000 Transfection Reagent (Thermo Fisher Scientific, 11668019) and 100 μl of Opti-MEM Reduced Serum Medium (Gibco, 31985062) in one well of a 12-well plate and incubated at room temperature for 20 min. A total of 60,000 cells were then added to each well, and the plate was placed in a cell culture incubator for 48 hours.

### NMR-based metabolomics

After harvesting mice prostates, DLP lobes were isolated and immediately flash frozen. Quantification of the levels of metabolites was performed using high-resolution magic angle spinning (54.7°) NMR, as described (*54*).

### Chromatin immunoprecipitation sequencing

Myc-CaP cells treated with 1 mM DMOG (Tocris; 4408) for 6 hours were incubated in 1% PFA (Electron Microscopy Sciences) for 8 min and quenched with 0.125 M glycine buffer for 5 min at room temperature. Cross-linked cells were resuspended in 500 μl of 10 mM Hepes, 60 mM KCl, 1 mM EDTA, 0.075% (v/v) NP40, 1 mM DTT, and 1 mM phenylmethylsulfonyl fluoride (pH 7.6) and incubated on ice for 5 min. After centrifugation at 400*g* for 5 min, nuclear pellets were resuspended in ChIP lysis buffer [50 mM tris-HCl (pH 8), 0.1% SDS, and 10 mM EDTA] and sonicated in 300- to 400–base pair (bp) DNA fragments (Covaris). Fifty micrograms of chromatin was immunoprecipitated in ChIP buffer [50 mM tris-HCl (pH 7.5), 140 mM NaCl, 1 mM EDTA, and 1% Triton X-100] using 5 μg of HIF1A antibody (CST 36169) or a rabbit IgG as negative control. Thirty microliters of Dynabeads Protein G (Thermo Fisher Scientific) was added to the immunocomplexes for 2 hours at 4°C. DNA was recovered by reverse cross-linking overnight at 65°C and extracted using phenol/chloroform/isoamyl alcohol. Precipitated DNA was purified using Agencourt AMPure XP beads (Beckman Coulter). ChIP-seq libraries were prepared using the MicroPlex Library Preparation Kit v2 (C05010014, Diagenode S.A., Seraing, Belgium) according to manufacturer’s instructions and sequenced using an Illumina HiSeq 4000 as single-end 50-bp reads. Raw sequencing data were mapped to the mm10 reference genome using Bowtie 2 (*55*), and MACS2 algorithm (*56*) was used for the peak calling with nonimmunoprecipitated chromatin as control. All peaks with a false discovery rate greater than 0.05 were excluded from further analysis.

### scRNA-seq and data analysis

Trypan blue exclusion assay was used to determine cell number and viability of FACS-sorted cells using a Neubauer Chamber. Cells with a viability of >95% were processed using the Chromium Controller (10X Genomics, Leiden, The Netherlands). Ten thousand cells were loaded per well to capture between 5000 and 10,000 cells in nanoliter-scale Gel Beads-in-Emulsion (GEMs). Chromium Single-Cell 3′ Reagent Kits (10X Genomics ref. CG00052 Rev. E) were used to generate single-cell 3′ mRNA-seq libraries. Briefly, barcoded gel beads were combined with an RT master mix containing cells and partitioning oil onto Chromium Chip A to generate GEMs. After complementary DNA (cDNA) synthesis and barcoding from poly-adenylated mRNA, GEMs were disrupted and pooled before the amplification of cDNA by 10 polymerase chain reaction (PCR) cycles. Sequencing libraries were constructed after enzymatic fragmentation and size selection, by the addition of Illumina P5 and P7 primers (Paris, France) and sample index via end repair, A tailing, adaptor ligation, and 12 cycles of PCR amplification. Quality control and quantification of libraries was performed using Bioanalyzer 2100 (Agilent Technologies, Santa Clara, CA). Generated libraries were sequenced on Illumina HiSeq 4000 as 100 bases paired-end reads. RTA 2.7.7 and Cell Ranger 3.0.1 mkfastq were used for image analysis, base calling, and demultiplexing. Cell Ranger 3.0.1 count and the mouse reference 3.0.0 (mm10 and Ensembl release 93) were used for alignment, barcode, and UMI (Unique Molecular Identifier) filtering and counting.

The Read10X function of the Seurat v 4 (*57*) R [version (v) 4.0.2] was used to read the output of the Cell Ranger pipeline and obtain a matrix of the number of reads of each gene detected in each cell. Genes expressed in less than 10 cells were excluded. Cells from the different samples with more than 100 and less than 7000 expressed genes and with lower than 20% mitochondrial genes were analyzed, as previously described (*17*).

### Trajectory inference

Read mapping, barcode, and UMI filtering and counting were performed using Cell Ranger (*58*) 3.0.1 count and mouse reference 3.0.0 (mm10 and Ensembl release 93). Aggregation of the corresponding results for the four samples, principal component analysis dimensionality reduction, and graph-based clustering were performed using Cell Ranger 3.0.1 aggr. The resulting filtered feature barcode matrix was processed using Seurat (*57*) 3.2.0 package: Cells with at least 100 and less than 5000 expressed genes and with less than 20% mitochondrial reads were retained, log normalization was performed, and only genes with at least five UMI in at least 10 cells were retained. Trajectory inference was performed on cells from clusters 10, 12, and 20 using dyno (*59*) 0.1.2 package and slingshot method (*60*), and milestone 3 was designated as root. Feature importance value (*59*) for each gene was calculated using calculate_overall_feature_importance from dyno 0.1.2 package.

### TCGA analysis

The correlation of the expression of various genes with that of HIF1A was analyzed in the PRAD cohort (*n* = 496 patients with PCa) of the TCGA. The correlation of TGM2 expression with the HIF1A gene signature was performed using GEPIA2 (*61*). Pearson’s correlation coefficient was used to determine the association between two genes, and Spearman’s correlation coefficient was used to determine the association between TGM2 and the various gene signatures. A significant direct correlation was defined as having a correlation coefficient of ≥0.2 and a *P* value of ≤0.05. UALCAN was used to analyze TGM2 levels in patients with PCa with different Gleason scores (*62*).

### Human prostate TMA (Tissue microarray)

All human samples and associated clinical data were collected with patient’s written informed consent and were anonymized before use. A total of 62 treatment-naïve patients with PCa who had undergone surgical prostatectomy between April 2009 and April 2014 were recruited. TMAs were constructed at the Center of Biological Resources at the University Hospital of Strasbourg from biopsies of patients with PCa (*n* = 62) with different pathological stages, ranging from intermediate (*n* = 34) to high grade (*n* = 28) (Gleason score 3 + 3 to 4 + 5, pT2a to pT3b), as well as their adjacent normal areas. Two cores of tumoral tissues (1.5-mm diameter) per patient were included in the TMAs.

Clinical information of the patients was collected in patients’ medical files (urology and oncology stations) by a research assistant technician under the responsibility of the University Hospital of Strasbourg. Of the 62 patients, 6 were excluded from the analyses as their clinical follow-up data were missing. The PFS is the time from prostatectomy until biochemical recurrence, defined as an increase in postsurgery prostate-specific antigen value by 0.2 ng/ml.

Immunohistochemical detection of TGM2 was performed as described above. Staining intensity was scored by an oncology resident, blinded to outcomes, from 0 to 2: 0 = negative, 1 = weak staining, and 2 = strong staining. Patients were classified into two groups according to TGM2 expression: weak/negative versus high. TGM2 expression was then associated with the patients’ PFS.

### Statistical analysis

Comparison between two groups was performed using a two-tailed Student’s *t* test, with *P* values less than 0.05, 0.01, and 0.001 depicted by *, **, and ***, respectively. Error bars represent SEM. The number of replicates is indicated in the figure legends. Gehan-Breslow-Wilcoxon test was used to determine the *P* value of the survival analysis. The Wilcoxon rank sum test was used to determine DEGs.

## Notes

View/request a protocol for this paper from *Bio-protocol*.

## References

[1] F. Bray, J. Ferlay, I. Soerjomataram, R. L. Siegel, L. A. Torre, A. Jemal, Global cancer statistics 2018: GLOBOCAN estimates of incidence and mortality worldwide for 36 cancers in 185 countries. CA Cancer J. Clin. 68, 394–424 (2018). 30207593 10.3322/caac.21492

[2] R. J. Rebello, C. Oing, K. E. Knudsen, S. Loeb, D. C. Johnson, R. E. Reiter, S. Gillessen, T. Van der Kwast, R. G. Bristow, Prostate cancer. Nat. Rev. Dis. Primers. 7, 9 (2021). 33542230 10.1038/s41572-020-00243-0

[3] G. Wang, D. Zhao, D. J. Spring, R. A. DePinho, Genetics and biology of prostate cancer. Genes Dev. 32, 1105–1140 (2018). 30181359 10.1101/gad.315739.118 PMC6120714

[4] M. M. Shen, C. Abate-Shen, Molecular genetics of prostate cancer: New prospects for old challenges. Genes Dev. 24, 1967–2000 (2010). 20844012 10.1101/gad.1965810 PMC2939361

[5] I. M. Thompson, P. J. Goodman, C. M. Tangen, M. S. Lucia, G. J. Miller, L. G. Ford, M. M. Lieber, R. D. Cespedes, J. N. Atkins, S. M. Lippman, S. M. Carlin, A. Ryan, C. M. Szczepanek, J. J. Crowley, C. A. Coltman Jr., The influence of finasteride on the development of prostate cancer. N. Engl. J. Med. 349, 215–224 (2003). 12824459 10.1056/NEJMoa030660

[6] D. M. Gilkes, G. L. Semenza, D. Wirtz, Hypoxia and the extracellular matrix: Drivers of tumour metastasis. Nat. Rev. Cancer 14, 430–439 (2014). 24827502 10.1038/nrc3726 PMC4283800

[7] A. J. Majmundar, W. J. Wong, M. C. Simon, Hypoxia-inducible factors and the response to hypoxic stress. Mol. Cell 40, 294–309 (2010). 20965423 10.1016/j.molcel.2010.09.022 PMC3143508

[8] L. Schito, G. L. Semenza, Hypoxia-inducible factors: Master regulators of cancer progression. Trends Cancer. 2, 758–770 (2016). 28741521 10.1016/j.trecan.2016.10.016

[9] G. L. Semenza, Targeting HIF-1 for cancer therapy. Nat. Rev. Cancer 3, 721–732 (2003). 13130303 10.1038/nrc1187

[10] A. Lekas, A. C. Lazaris, C. Deliveliotis, M. Chrisofos, C. Zoubouli, D. Lapas, T. Papathomas, I. Fokitis, L. Nakopoulou, The expression of hypoxia-inducible factor-1alpha (HIF-1alpha) and angiogenesis markers in hyperplastic and malignant prostate tissue. Anticancer Res 26, 2989–2993 (2006). 16886625

[11] D. M. Carnell, R. E. Smith, F. M. Daley, M. I. Saunders, S. M. Bentzen, P. J. Hoskin, An immunohistochemical assessment of hypoxia in prostate carcinoma using pimonidazole: Implications for radioresistance. Int. J. Radiat. Oncol. Biol. Phys. 65, 91–99 (2006). 16563659 10.1016/j.ijrobp.2005.11.044

[12] C. Parker, M. Milosevic, A. Toi, J. Sweet, T. Panzarella, R. Bristow, C. Catton, P. Catton, J. Crook, M. Gospodarowicz, M. McLean, P. Warde, R. P. Hill, Polarographic electrode study of tumor oxygenation in clinically localized prostate cancer. Int. J. Radiat. Oncol. Biol. Phys. 58, 750–757 (2004). 14967430 10.1016/S0360-3016(03)01621-3

[13] R. Vergis, C. M. Corbishley, A. R. Norman, J. Bartlett, S. Jhavar, M. Borre, S. Heeboll, A. Horwich, R. Huddart, V. Khoo, R. Eeles, C. Cooper, M. Sydes, D. Dearnaley, C. Parker, Intrinsic markers of tumour hypoxia and angiogenesis in localised prostate cancer and outcome of radical treatment: A retrospective analysis of two randomised radiotherapy trials and one surgical cohort study. Lancet Oncol. 9, 342–351 (2008). 18343725 10.1016/S1470-2045(08)70076-7

[14] H. Zhong, G. L. Semenza, J. W. Simons, A. M. De Marzo, Up-regulation of hypoxia-inducible factor 1alpha is an early event in prostate carcinogenesis. Cancer Detect. Prev. 28, 88–93 (2004). 15068831 10.1016/j.cdp.2003.12.009

[15] C. K. Ratnacaram, M. Teletin, M. Jiang, X. Meng, P. Chambon, D. Metzger, Temporally controlled ablation of PTEN in adult mouse prostate epithelium generates a model of invasive prostatic adenocarcinoma. Proc. Natl. Acad. Sci. U.S.A. 105, 2521–2526 (2008). 18268330 10.1073/pnas.0712021105 PMC2268169

[16] M. Parisotto, E. Grelet, R. El Bizri, Y. Dai, J. Terzic, D. Eckert, L. Gargowitsch, J. M. Bornert, D. Metzger, PTEN deletion in luminal cells of mature prostate induces replication stress and senescence in vivo. J. Exp. Med. 215, 1749–1763 (2018). 29743291 10.1084/jem.20171207 PMC5987915

[17] M. A. Abu El Maaty, E. Grelet, C. Keime, A. I. Rerra, J. Gantzer, C. Emprou, J. Terzic, R. Lutzing, J. M. Bornert, G. Laverny, D. Metzger, Single-cell analyses unravel cell type-specific responses to a vitamin D analog in prostatic precancerous lesions. Sci. Adv. 7, eabg5982 (2021). 34330705 10.1126/sciadv.abg5982 PMC8324049

[18] R. Tibes, G. S. Falchook, D. D. Von Hoff, G. J. Weiss, T. Iyengar, R. Kurzrock, L. Pestano, A. M. Lowe, R. S. Herbst, Results from a phase I, dose-escalation study of PX-478, an orally available inhibitor of HIF-1α. J. Clin. Oncol. 28, 3076 (2010).20479403

[19] S. Welsh, R. Williams, L. Kirkpatrick, G. Paine-Murrieta, G. Powis, Antitumor activity and pharmacodynamic properties of PX-478, an inhibitor of hypoxia-inducible factor-1alpha. Mol. Cancer Ther. 3, 233–244 (2004). 15026543

[20] S. T. Palayoor, J. B. Mitchell, D. Cerna, W. Degraff, M. John-Aryankalayil, C. N. Coleman, PX-478, an inhibitor of hypoxia-inducible factor-1alpha, enhances radiosensitivity of prostate carcinoma cells. Int. J. Cancer 123, 2430–2437 (2008). 18729192 10.1002/ijc.23807 PMC4277812

[21] H. Guo, X. Ci, M. Ahmed, J. T. Hua, F. Soares, D. Lin, L. Puca, A. Vosoughi, H. Xue, E. Li, P. Su, S. Chen, T. Nguyen, Y. Liang, Y. Zhang, X. Xu, J. Xu, A. V. Sheahan, W. Ba-Alawi, S. Zhang, O. Mahamud, R. N. Vellanki, M. Gleave, R. G. Bristow, B. Haibe-Kains, J. T. Poirier, C. M. Rudin, M. S. Tsao, B. G. Wouters, L. Fazli, F. Y. Feng, L. Ellis, T. van der Kwast, A. Berlin, M. Koritzinsky, P. C. Boutros, A. Zoubeidi, H. Beltran, Y. Wang, H. H. He, ONECUT2 is a driver of neuroendocrine prostate cancer. Nat. Commun. 10, 278 (2019). 30655535 10.1038/s41467-018-08133-6 PMC6336817

[22] S. Mei, Q. Qin, Q. Wu, H. Sun, R. Zheng, C. Zang, M. Zhu, J. Wu, X. Shi, L. Taing, T. Liu, M. Brown, C. A. Meyer, X. S. Liu, Cistrome Data Browser: A data portal for ChIP-Seq and chromatin accessibility data in human and mouse. Nucleic Acids Res. 45, D658–D662 (2017). 27789702 10.1093/nar/gkw983 PMC5210658

[23] B. H. Li, M. A. Garstka, Z. F. Li, Chemokines and their receptors promoting the recruitment of myeloid-derived suppressor cells into the tumor. Mol. Immunol. 117, 201–215 (2020). 31835202 10.1016/j.molimm.2019.11.014

[24] B. Wang, J. Kohli, M. Demaria, Senescent cells in cancer therapy: Friends or foes? Trends Cancer. 6, 838–857 (2020). 32482536 10.1016/j.trecan.2020.05.004

[25] Y. Nakanuma, M. Sasaki, K. Harada, Autophagy and senescence in fibrosing cholangiopathies. J. Hepatol. 62, 934–945 (2015). 25435435 10.1016/j.jhep.2014.11.027

[26] B. Mirlekar, Y. Pylayeva-Gupta, IL-12 family cytokines in cancer and immunotherapy. Cancers 13, 167 (2021). 33418929 10.3390/cancers13020167 PMC7825035

[27] P. Mu, Z. Zhang, M. Benelli, W. R. Karthaus, E. Hoover, C. C. Chen, J. Wongvipat, S. Y. Ku, D. Gao, Z. Cao, N. Shah, E. J. Adams, W. Abida, P. A. Watson, D. Prandi, C. H. Huang, E. de Stanchina, S. W. Lowe, L. Ellis, H. Beltran, M. A. Rubin, D. W. Goodrich, F. Demichelis, C. L. Sawyers, SOX2 promotes lineage plasticity and antiandrogen resistance in TP53- and RB1-deficient prostate cancer. Science 355, 84–88 (2017). 28059768 10.1126/science.aah4307 PMC5247742

[28] S. Y. Ku, S. Rosario, Y. Wang, P. Mu, M. Seshadri, Z. W. Goodrich, M. M. Goodrich, D. P. Labbe, E. C. Gomez, J. Wang, H. W. Long, B. Xu, M. Brown, M. Loda, C. L. Sawyers, L. Ellis, D. W. Goodrich, Rb1 and Trp53 cooperate to suppress prostate cancer lineage plasticity, metastasis, and antiandrogen resistance. Science 355, 78–83 (2017). 28059767 10.1126/science.aah4199 PMC5367887

[29] H. Arase, T. Saito, J. H. Phillips, L. L. Lanier, Cutting edge: The mouse NK cell-associated antigen recognized by DX5 monoclonal antibody is CD49b (alpha 2 integrin, very late antigen-2). J. Immunol. 167, 1141–1144 (2001). 11466327 10.4049/jimmunol.167.3.1141

[30] D. H. Kong, Y. K. Kim, M. R. Kim, J. H. Jang, S. Lee, Emerging roles of vascular cell adhesion molecule-1 (VCAM-1) in immunological disorders and cancer. Int. J. Mol. Sci. 19, 1057 (2018). 29614819 10.3390/ijms19041057 PMC5979609

[31] R. Tempest, S. Guarnerio, R. Maani, J. Cooper, N. Peake, The biological and biomechanical role of transglutaminase-2 in the tumour microenvironment. Cancers 13, 2788 (2021). 34205140 10.3390/cancers13112788 PMC8199963

[32] H. B. Ragnum, L. Vlatkovic, A. K. Lie, K. Axcrona, C. H. Julin, K. M. Frikstad, K. H. Hole, T. Seierstad, H. Lyng, The tumour hypoxia marker pimonidazole reflects a transcriptional programme associated with aggressive prostate cancer. Br. J. Cancer 112, 382–390 (2015). 25461803 10.1038/bjc.2014.604 PMC4453458

[33] A. Liberzon, C. Birger, H. Thorvaldsdottir, M. Ghandi, J. P. Mesirov, P. Tamayo, The Molecular Signatures Database (MSigDB) hallmark gene set collection. Cell Syst. 1, 417–425 (2015). 26771021 10.1016/j.cels.2015.12.004 PMC4707969

[34] N. C. Denko, Hypoxia, HIF1 and glucose metabolism in the solid tumour. Nat. Rev. Cancer 8, 705–713 (2008). 19143055 10.1038/nrc2468

[35] L. A. Begley, S. Kasina, R. Mehra, S. Adsule, A. J. Admon, R. J. Lonigro, A. M. Chinnaiyan, J. A. Macoska, CXCL5 promotes prostate cancer progression. Neoplasia 10, 244–254 (2008). 18320069 10.1593/neo.07976 PMC2262133

[36] T. O. Adekoya, R. M. Richardson, Cytokines and chemokines as mediators of prostate cancer metastasis. Int. J. Mol. Sci. 21, 4449 (2020). 32585812 10.3390/ijms21124449 PMC7352203

[37] G. Wang, X. Lu, P. Dey, P. Deng, C. C. Wu, S. Jiang, Z. Fang, K. Zhao, R. Konaparthi, S. Hua, J. Zhang, E. M. Li-Ning-Tapia, A. Kapoor, C. J. Wu, N. B. Patel, Z. Guo, V. Ramamoorthy, T. N. Tieu, T. Heffernan, D. Zhao, X. Shang, S. Khadka, P. Hou, B. Hu, E. J. Jin, W. Yao, X. Pan, Z. Ding, Y. Shi, L. Li, Q. Chang, P. Troncoso, C. J. Logothetis, M. J. McArthur, L. Chin, Y. A. Wang, R. A. DePinho, Targeting YAP-dependent MDSC infiltration impairs tumor progression. Cancer Discov. 6, 80–95 (2016). 26701088 10.1158/2159-8290.CD-15-0224 PMC4707102

[38] A. Calcinotto, C. Spataro, E. Zagato, D. Di Mitri, V. Gil, M. Crespo, G. De Bernardis, M. Losa, M. Mirenda, E. Pasquini, A. Rinaldi, S. Sumanasuriya, M. B. Lambros, A. Neeb, R. Luciano, C. A. Bravi, D. Nava-Rodrigues, D. Dolling, T. Prayer-Galetti, A. Ferreira, A. Briganti, A. Esposito, S. Barry, W. Yuan, A. Sharp, J. de Bono, A. Alimonti, IL-23 secreted by myeloid cells drives castration-resistant prostate cancer. Nature 559, 363–369 (2018). 29950727 10.1038/s41586-018-0266-0 PMC6461206

[39] Z. Husain, Y. Huang, P. Seth, V. P. Sukhatme, Tumor-derived lactate modifies antitumor immune response: Effect on myeloid-derived suppressor cells and NK cells. J. Immunol. 191, 1486–1495 (2013). 23817426 10.4049/jimmunol.1202702

[40] J. Stultz, L. Fong, How to turn up the heat on the cold immune microenvironment of metastatic prostate cancer. Prostate Cancer Prostatic Dis. 24, 697–717 (2021). 33820953 10.1038/s41391-021-00340-5 PMC8384622

[41] R. Y. Ebright, M. A. Zachariah, D. S. Micalizzi, B. S. Wittner, K. L. Niederhoffer, L. T. Nieman, B. Chirn, D. F. Wiley, B. Wesley, B. Shaw, E. Nieblas-Bedolla, L. Atlas, A. Szabolcs, A. J. Iafrate, M. Toner, D. T. Ting, P. K. Brastianos, D. A. Haber, S. Maheswaran, HIF1A signaling selectively supports proliferation of breast cancer in the brain. Nat. Commun. 11, 6311 (2020). 33298946 10.1038/s41467-020-20144-w PMC7725834

[42] R. L. Eckert, Transglutaminase 2 takes center stage as a cancer cell survival factor and therapy target. Mol. Carcinog. 58, 837–853 (2019). 30693974 10.1002/mc.22986 PMC7754084

[43] G. Y. Jang, J. H. Jeon, S. Y. Cho, D. M. Shin, C. W. Kim, E. M. Jeong, H. C. Bae, T. W. Kim, S. H. Lee, Y. Choi, D. S. Lee, S. C. Park, I. G. Kim, Transglutaminase 2 suppresses apoptosis by modulating caspase 3 and NF-κB activity in hypoxic tumor cells. Oncogene 29, 356–367 (2010). 19838207 10.1038/onc.2009.342

[44] S. Kumar, K. Mehta, Tissue transglutaminase constitutively activates HIF-1α promoter and nuclear Factor-κB via a non-canonical pathway. PLOS ONE 7, e49321 (2012). 23185316 10.1371/journal.pone.0049321 PMC3501523

[45] A. L. Han, S. Kumar, J. Y. Fok, A. K. Tyagi, K. Mehta, Tissue transglutaminase expression promotes castration-resistant phenotype and transcriptional repression of androgen receptor. Eur. J. Cancer 50, 1685–1696 (2014). 24656569 10.1016/j.ejca.2014.02.014

[46] J. J. Jacoby, B. Erez, M. V. Korshunova, R. R. Williams, K. Furutani, O. Takahashi, L. Kirkpatrick, S. M. Lippman, G. Powis, M. S. O’Reilly, R. S. Herbst, Treatment with HIF-1alpha antagonist PX-478 inhibits progression and spread of orthotopic human small cell lung cancer and lung adenocarcinoma in mice. J. Thorac. Oncol. 5, 940–949 (2010). 20512076 10.1097/JTO.0b013e3181dc211f PMC3782111

[47] C. Parker, E. Castro, K. Fizazi, A. Heidenreich, P. Ost, G. Procopio, B. Tombal, S. Gillessen; ESMO Guidelines Committee. Electronic address: clinicalguidelines@esmo.org, Prostate cancer: ESMO clinical practice guidelines for diagnosis, treatment and follow-up. Ann Oncol. 31, 1119–1134 (2020). 32593798 10.1016/j.annonc.2020.06.011

[48] K. Sarkar, Z. Cai, R. Gupta, N. Parajuli, K. Fox-Talbot, M. S. Darshan, F. J. Gonzalez, G. L. Semenza, Hypoxia-inducible factor 1 transcriptional activity in endothelial cells is required for acute phase cardioprotection induced by ischemic preconditioning. Proc. Natl. Acad. Sci. U.S.A. 109, 10504–10509 (2012). 22699503 10.1073/pnas.1208314109 PMC3387090

[49] P. Bankhead, M. B. Loughrey, J. A. Fernandez, Y. Dombrowski, D. G. McArt, P. D. Dunne, S. McQuaid, R. T. Gray, L. J. Murray, H. G. Coleman, J. A. James, M. Salto-Tellez, P. W. Hamilton, QuPath: Open source software for digital pathology image analysis. Sci. Rep. 7, 16878 (2017). 29203879 10.1038/s41598-017-17204-5 PMC5715110

[50] R. U. Lukacs, A. S. Goldstein, D. A. Lawson, D. Cheng, O. N. Witte, Isolation, cultivation and characterization of adult murine prostate stem cells. Nat. Protoc. 5, 702–713 (2010). 20360765 10.1038/nprot.2010.11 PMC2943378

[51] L. Sackmann Sala, F. Boutillon, G. Menara, A. De Goyon-Pelard, M. Leprevost, J. Codzamanian, N. Lister, J. Pencik, A. Clark, N. Cagnard, C. Bole-Feysot, R. Moriggl, G. P. Risbridger, R. A. Taylor, L. Kenner, J. E. Guidotti, V. Goffin, A rare castration-resistant progenitor cell population is highly enriched in Pten-null prostate tumours. J. Pathol. 243, 51–64 (2017). 28603917 10.1002/path.4924

[52] J. Drost, W. R. Karthaus, D. Gao, E. Driehuis, C. L. Sawyers, Y. Chen, H. Clevers, Organoid culture systems for prostate epithelial and cancer tissue. Nat. Protoc. 11, 347–358 (2016). 26797458 10.1038/nprot.2016.006 PMC4793718

[53] W. R. Karthaus, P. J. Iaquinta, J. Drost, A. Gracanin, R. van Boxtel, J. Wongvipat, C. M. Dowling, D. Gao, H. Begthel, N. Sachs, R. G. J. Vries, E. Cuppen, Y. Chen, C. L. Sawyers, H. C. Clevers, Identification of multipotent luminal progenitor cells in human prostate organoid cultures. Cell 159, 163–175 (2014). 25201529 10.1016/j.cell.2014.08.017 PMC4772677

[54] L. Bender, F. Somme, E. Ruhland, A. E. Cicek, C. Bund, I. J. Namer, Metabolomic profile of aggressive meningiomas by using high-resolution magic angle spinning nuclear magnetic resonance. J. Proteome Res. 19, 292–299 (2020). 31679342 10.1021/acs.jproteome.9b00521

[55] B. Langmead, S. L. Salzberg, Fast gapped-read alignment with Bowtie 2. Nat. Methods 9, 357–359 (2012). 22388286 10.1038/nmeth.1923 PMC3322381

[56] Y. Zhang, T. Liu, C. A. Meyer, J. Eeckhoute, D. S. Johnson, B. E. Bernstein, C. Nusbaum, R. M. Myers, M. Brown, W. Li, X. S. Liu, Model-based analysis of ChIP-Seq (MACS). Genome Biol. 9, R137 (2008). 18798982 10.1186/gb-2008-9-9-r137 PMC2592715

[57] T. Stuart, A. Butler, P. Hoffman, C. Hafemeister, E. Papalexi, W. M. Mauck III, Y. Hao, M. Stoeckius, P. Smibert, R. Satija, Comprehensive integration of single-cell data. Cell 177, 1888–1902.e21 (2019). 31178118 10.1016/j.cell.2019.05.031 PMC6687398

[58] G. X. Zheng, J. M. Terry, P. Belgrader, P. Ryvkin, Z. W. Bent, R. Wilson, S. B. Ziraldo, T. D. Wheeler, G. P. McDermott, J. Zhu, M. T. Gregory, J. Shuga, L. Montesclaros, J. G. Underwood, D. A. Masquelier, S. Y. Nishimura, M. Schnall-Levin, P. W. Wyatt, C. M. Hindson, R. Bharadwaj, A. Wong, K. D. Ness, L. W. Beppu, H. J. Deeg, C. McFarland, K. R. Loeb, W. J. Valente, N. G. Ericson, E. A. Stevens, J. P. Radich, T. S. Mikkelsen, B. J. Hindson, J. H. Bielas, Massively parallel digital transcriptional profiling of single cells. Nat. Commun. 8, 14049 (2017). 28091601 10.1038/ncomms14049 PMC5241818

[59] W. Saelens, R. Cannoodt, H. Todorov, Y. Saeys, A comparison of single-cell trajectory inference methods. Nat. Biotechnol. 37, 547–554 (2019). 30936559 10.1038/s41587-019-0071-9

[60] K. Street, D. Risso, R. B. Fletcher, D. Das, J. Ngai, N. Yosef, E. Purdom, S. Dudoit, Slingshot: Cell lineage and pseudotime inference for single-cell transcriptomics. BMC Genomics 19, 477 (2018). 29914354 10.1186/s12864-018-4772-0 PMC6007078

[61] Z. Tang, B. Kang, C. Li, T. Chen, Z. Zhang, GEPIA2: An enhanced web server for large-scale expression profiling and interactive analysis. Nucleic Acids Res. 47, W556–W560 (2019). 31114875 10.1093/nar/gkz430 PMC6602440

[62] D. S. Chandrashekar, B. Bashel, S. A. H. Balasubramanya, C. J. Creighton, I. Ponce-Rodriguez, B. Chakravarthi, S. Varambally, UALCAN: A portal for facilitating tumor subgroup gene expression and survival analyses. Neoplasia 19, 649–658 (2017). 28732212 10.1016/j.neo.2017.05.002 PMC5516091

