# Supplementary material for: Hypoxia-mediated stabilization of HIF1A in prostatic intraepithelial neoplasia promotes cell plasticity and malignant progression
Source: Sci Adv. 2022 Jul 22;8(29):eabo2295. doi: 10.1126/sciadv.abo2295 (PMC9307253; doi:10.1126/sciadv.abo2295)
Supplement: Supplementary file 1 — Figs. S1 to S6 Table S13 [file sciadv.abo2295_sm.pdf]

Science Advances
AAAS

Supplementary Materials for

Hypoxia-mediated stabilization of HIF1A in prostatic intraepithelial neoplasia promotes cell plasticity and malignant progression

Mohamed A. Abu el Maaty *et al.*

Corresponding author: Daniel Metzger, metzger@igbmc.fr; Gilles Laverny, laverny@igbmc.fr

Sci. Adv. **8**, eabo2295 (2022)
DOI: 10.1126/sciadv.abo2295

The PDF file includes:

Figs. S1 to S6
Legends for tables S1 to S12
Table S13

Other Supplementary Material for this manuscript includes the following:

Tables S1 to S12

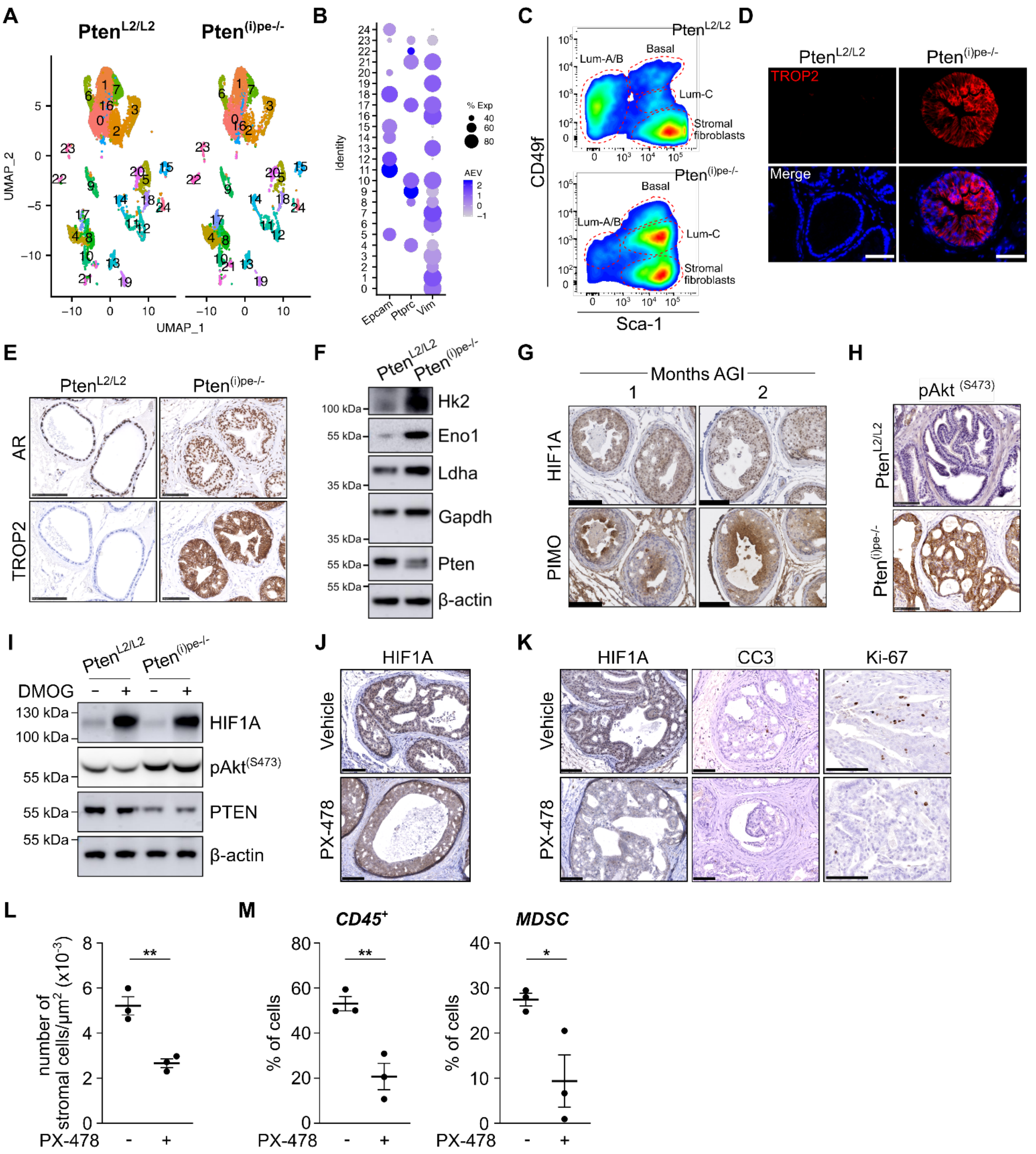

Fig. S1. (A) UMAP of cells of Pten$^{L2/L2}$ and Pten$^{(i)pe-/-}$ prostates at 3 months AGI. (B) Dot plot depicting the cell lineage-specific markers in these clusters. Dot size and color represent percentage of marker gene expression (% Exp) and the averaged expression value (AEV), respectively. Data presented are of combined Pten$^{L2/L2}$ and Pten$^{(i)pe-/-}$ cells. (C) Pseudocolor FACS

plots depicting the abundance of the different epithelial subsets and stromal fibroblasts in $Pten^{L2/L2}$ and $Pten^{(i)pe-/-}$ prostates at 3 months AGI. (D) Representative immunofluorescence detection of TROP2-positive cells (red) in $Pten^{L2/L2}$ and $Pten^{(i)pe-/-}$ prostates at 3 months AGI. Representative images of the DLP are shown. N=3 mice/condition. DAPI (blue); scale bars: 50 µm. (E) Representative immunohistochemical detection of AR and TROP2 in the DLP of $Pten^{L2/L2}$ and $Pten^{(i)pe-/-}$ mice at 3 months AGI. N=3 mice/condition. Scale bars: 100 µm. (F) Western blot analysis of HIF1A target genes in prostate extracts of $Pten^{L2/L2}$ and $Pten^{(i)pe-/-}$ mice at 3 months AGI. Lysates of 3 prostates per condition were pooled. β-actin was used as a loading control. (G) Representative immunohistochemical detection of HIF1A and pimonidazole (PIMO) in the DLP of $Pten^{(i)pe-/-}$ mice at 1 and 2 months AGI. N=3 mice/condition. Scale bars: 100 µm. (H) Representative immunohistochemical detection of phosphorylated Akt (S473) in the DLP of $Pten^{L2/L2}$ and $Pten^{(i)pe-/-}$ mice at 3 months AGI. N=3 mice/condition. Scale bars: 100 µm. (I) Western blot analysis of HIF1A levels in organoids generated from prostates of $Pten^{L2/L2}$ and $Pten^{(i)pe-/-}$ mice at 3 months AGI, and cultured under normoxic conditions. Organoids treated for 6 h with DMOG were used as a positive control of HIF1A expression. β-actin was used as a loading control. (J) Representative immunohistochemical detection of HIF1A in the DLP of $Pten^{(i)pe-/-}$ mice treated at 3 months AGI with vehicle or PX-478 for 5 days. N=3 mice/condition. Scale bars: 100 µm. (K) Representative immunohistochemical detection of HIF1A, cleaved caspase 3 (CC3) and Ki-67 in the DLP of $Pten^{(i)pe-/-}$ mice treated with PX-478 at 10 months AGI. N=3 mice/condition. Scale bars: 100 µm. (L) Quantification of stromal cells in the DLP of $Pten^{(i)pe-/-}$ mice treated with vehicle or PX-478 at 10 months AGI. N=3 mice/condition. (M) Flow cytometric quantification of leukocytes (CD45+) and MDSCs in prostates of $Pten^{(i)pe-/-}$ mice treated with vehicle or PX-478 at 10 months AGI. N=3 mice/condition. *, $p < 0.05$; **, $p < 0.01$ calculated using a two-tailed t-test.

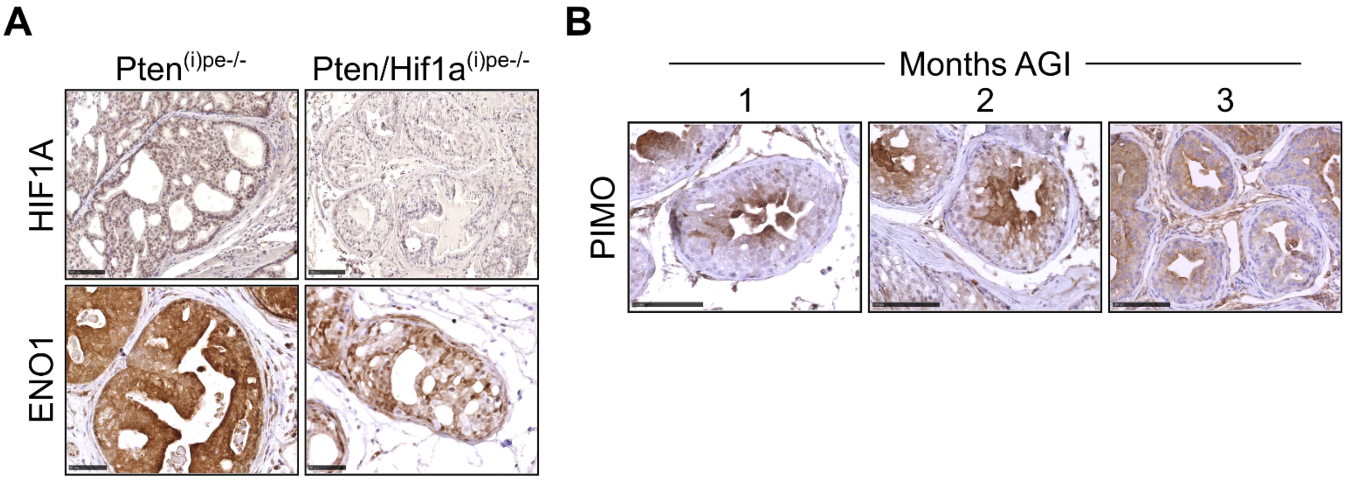

Fig. S2. (A) Representative immunohistochemical detection of HIF1A and ENO1 in the DLP of Pten$^{(i)pe-/-}$ and Pten/Hif1a$^{(i)pe-/-}$ mice at 3 months AGI. N=3 mice/condition. Scale bars: 100 µm. (B) Representative immunohistochemical detection of pimonidazole (PIMO) in the DLP of Pten/Hif1a$^{(i)pe-/-}$ mice at 1, 2 and 3 months AGI.

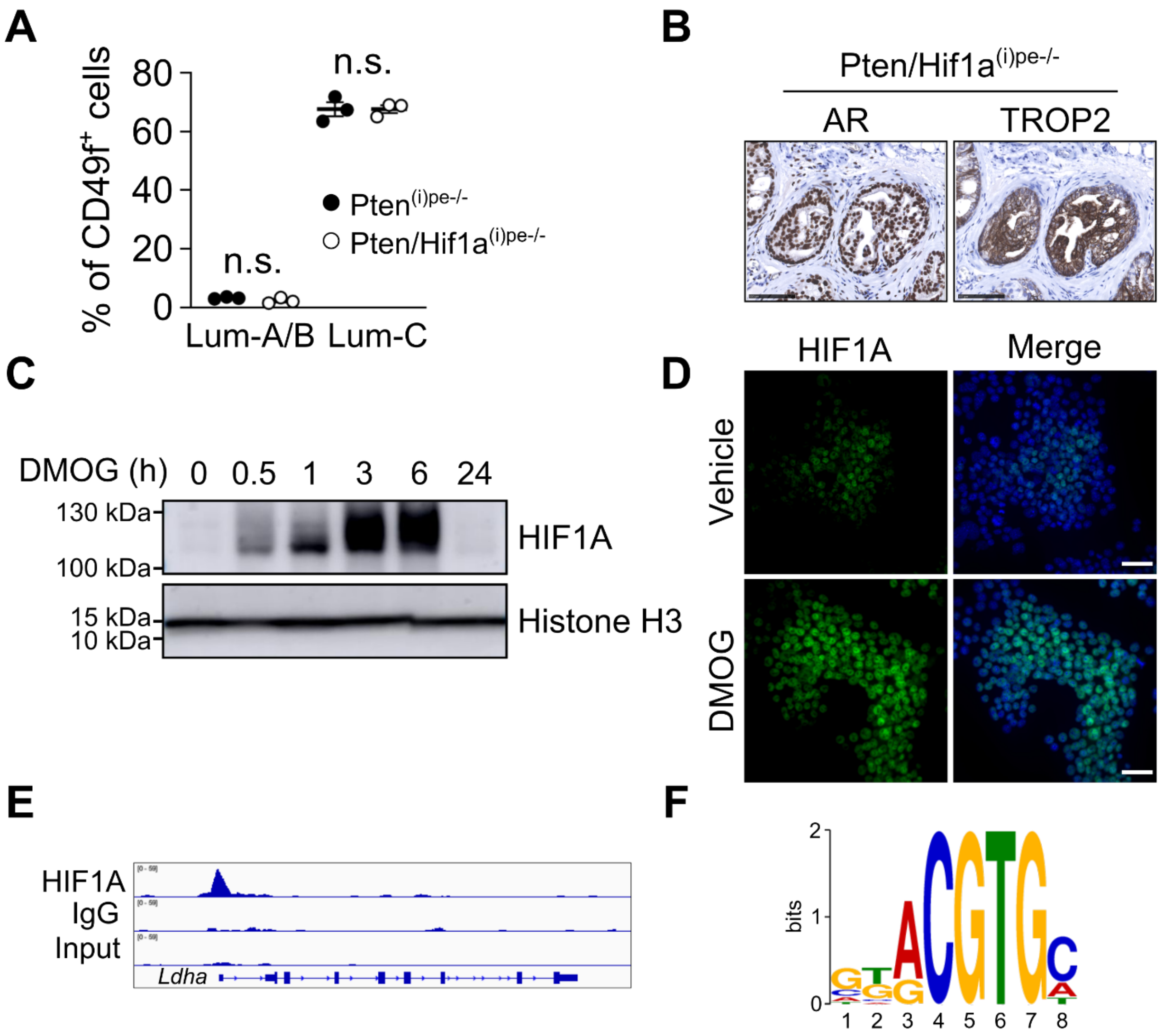

Fig. S3. (A) Quantification of luminal subsets in Pten$^{(i)pe-/-}$ and Pten/Hif1a$^{(i)pe-/-}$ mice at 3 months AGI by flow cytometry. N=3 mice/condition. Comparisons between groups were performed using a two-tailed t-test. n.s., $p \geq 0.05$. (B) Representative immunohistochemical detection of AR and TROP2 in the DLP of Pten/Hif1a$^{(i)pe-/-}$ mice at 3 months AGI. N=3 mice/condition. Scale bars: 100 µm. (C) Western blot analysis of HIF1A levels in Myc-CaP cells treated with DMOG for 0.5-24 h. Histone H3 was used as a loading control. (D) Representative immunofluorescent staining of HIF1A (green) and DAPI (blue) in Myc-CaP cells treated for 6 h with DMOG. Scale bar: 50 µm. (E) Representative Integrative Genomics Viewer track showing HIF1A DNA binding in the *Ldha* gene, as well as IgG- and non-immunoprecipitated DNA (input). (F) Hypoxia response element identified by the analysis of HIF1A binding sites in Myc-CaP cells using the Multiple Expectation maximizations for Motif Elicitation (MEME) tool.

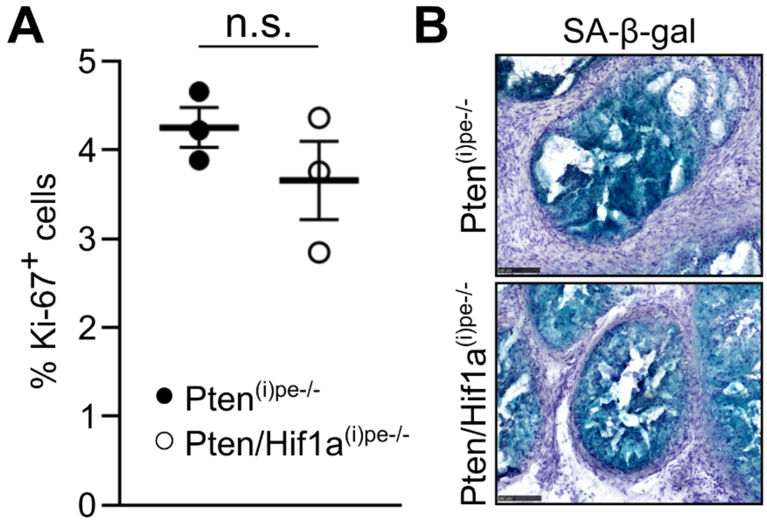

Fig. S4. Proliferation index (Ki-67$^+$ epithelial cells) (A) and SA-β-gal staining on prostatic sections of Pten$^{(i)pe-/-}$ and Pten/Hif1a$^{(i)pe-/-}$ mice at 3 months AGI (B). Representative images of the DLP are shown. N=3 mice/condition. n.s., $p \geq 0.05$ calculated using a two-tailed t-test. Scale bar: 100 μm.

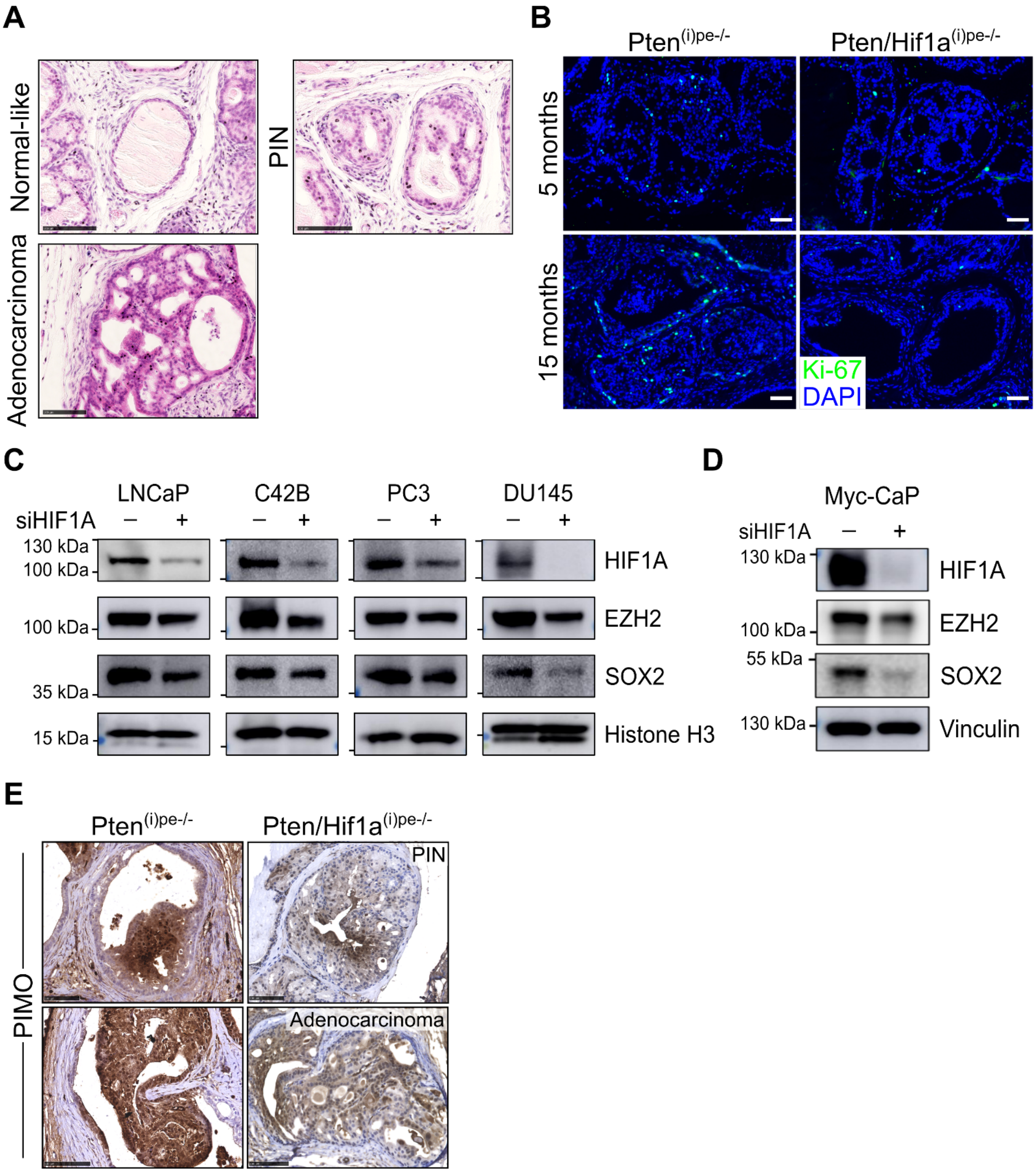

Fig. S5. (A) Representative images of histological scoring of glands in the DLP of $Pten^{(i)pe-/-}$ and $Pten/Hif1a^{(i)pe-/-}$ mice. Scale bars: 100 µm. (B) Representative immunodetection of Ki-67-positive cells (green) in the DLP of $Pten^{(i)pe-/-}$ and $Pten/Hif1a^{(i)pe-/-}$ mice at 5 and 15 months AGI. DAPI

(blue); scale bars: 100 μm. Western blot analysis of EZH2 and SOX2 in human (C) and mouse (D) PCa cell lines cultured under normoxic conditions, with or without HIF1A knockdown. Histone H3 and vinculin were used as loading controls. (E) Representative immunohistochemical detection of pimonidazole (PIMO) in the DLP of $Pten^{(i)pe-/-}$ and $Pten/Hif1a^{(i)pe-/-}$ mice at 15 months AGI. Scale bars: 100 μm.

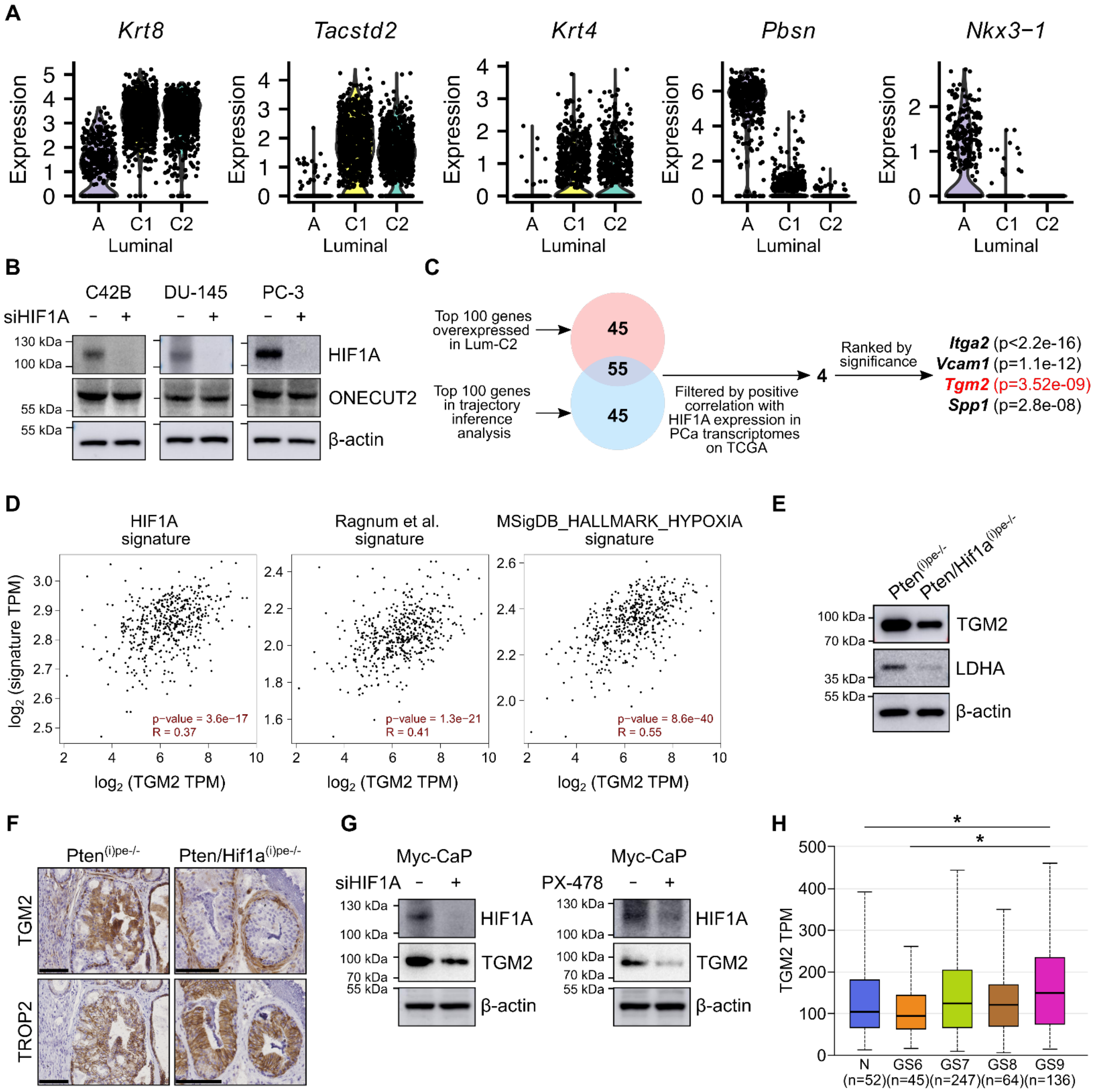

Fig. S6. (A) Violin plots depicting transcript levels of luminal-C markers (*Krt8*, *Tacstd2* and *Krt4*) and AR target genes (*Pbsn* and *Nkx3-1*) in luminal-A, -C1 and -C2 cells. (B) Western blot analysis of ONECUT2 in PCa cell lines with or without HIF1A knockdown, cultured under normoxic conditions. β-actin was used as a loading control. (C) Overview of the strategy to identify DEGs in luminal-C2 cells that correlate with HIF1A-driven PIN progression. (D) Analysis of the

correlation between TGM2 transcript levels and a HIF1A gene signature (*SLC2A1, HK2, PFKA, ALDOA, GAPDH, ENO1, LDHA, CA9, BNIP3* and *VEGFA*), a PCa-specific hypoxia signature (32), and a general hypoxia signature (33) in human PCa transcriptomes on the TCGA. Spearman correlation coefficient was used to determine the association between TGM2 expression and the investigated gene signatures. TPM: Transcripts per million. (E) Western blot analysis of TGM2 and the HIF1A target gene LDHA in FACS-sorted luminal-C cells of $Pten^{(i)pe-/-}$ and $Pten/Hif1a^{(i)pe-/-}$ mice at 3 months AGI. β-actin was used as a loading control. Lysates were obtained from a pool of 3 mice/condition. (F) Representative immunohistochemical detection of TGM2 and TROP2 in the DLP of $Pten^{(i)pe-/-}$ and $Pten/Hif1a^{(i)pe-/-}$ mice at 10 months AGI. Scale bars: 100 µm. (G) Western blot analysis of TGM2 in Myc-CaP cells in response to genetic and pharmacological inhibition of HIF1A, cultured under normoxic conditions. β-actin was used as a loading control. Cells were treated for 24 h with PX-478 at a final concentration of 50 µM. (H) Box plot depicting TGM2 transcript levels in human PCa patients of different Gleason scores (GS). Transcriptomes were obtained from the PRAD cohort of the TCGA. N=normal prostates. *, $p < 0.05$ calculated using a t-test.

Table S1. Gene signatures of the different clusters identified by scRNA-seq of $Pten^{L2/L2}$ and $Pten^{(i)pe-/-}$ prostates at 3 months AGI.

Table S2. List of DEGs obtained from the comparison of prostatic luminal cells (pool of luminal-A and -C) of $Pten^{(i)pe-/-}$ versus $Pten^{L2/L2}$ prostates.

Table S3. KEGG pathway analysis of the genes upregulated in prostatic luminal cells of $Pten^{(i)pe-/-}$ mice.

Table S4. List of DEGs obtained from the comparison of the different cell clusters of $Pten^{(i)pe-/-}$ versus $Pten^{L2/L2}$ prostates.

Table S5. KEGG pathway analysis of the genes upregulated in the different cell clusters of $Pten^{(i)pe-/-}$ versus $Pten^{L2/L2}$ prostates.

Table S6. Gene signatures of the different clusters identified by scRNA-seq of $Pten/Hif1a^{(i)pe-/-}$ prostates at 3 months AGI.

Table S7. List of DEGs obtained from the comparison of the different cell clusters of $Pten^{(i)pe-/-}$ versus $Pten/Hif1a^{(i)pe-/-}$ prostates.

Table S8. KEGG pathway analysis of the genes downregulated in the different cell clusters of $Pten/Hif1a^{(i)pe-/-}$ versus $Pten^{(i)pe-/-}$ prostates.

Table S9. List of metabolites measured by NMR in DLP of $Pten^{(i)pe-/-}$ and $Pten/Hif1a^{(i)pe-/-}$ mice at 3 months AGI.

Table S10. List of HIF1A target genes in Myc-CaP cells treated with DMOG identified by ChIP-seq.

Table S11. Number of cells in the different luminal subsets in $Pten^{(i)pe-/-}$ mice at 3, 6, 9 and 15 months AGI determined by scRNA-seq.

Table S12. Genes overexpressed in luminal-C2 cells compared to luminal-A and luminal-C1 cells obtained from scRNA-seq of $Pten^{(i)pe-/-}$ prostates at 3, 6, 9 and 15 months AGI.

		TGM2 low/negative n=25	TGM2 high n=31
Age (years)	Median	60.5	65
	Range	51-72	51-74
	Mean (SD)	62.9 (5.4)	63.2 (7.2) #
PSA at diagnosis (ng/mL)	Median	8.0	7.12
	Range	3.7-28.73	3.4-24.77
	Mean (SD)	9.2 (5.5)	8.7 (5.1) #
Gleason score	Median	7	7
	Range	7-9	7-9
	Mean (SD)	7.4 (0.7)	7.4 (0.7) #
Follow-up (months)	Median	108	104
	Range	94-119	82-168
	Mean (SD)	103.8 (8.2)	105.8 (16.4) #
PFS (months)	Median	103	79
	Range	20-119	20-120
	Mean (SD)	87.7 (29.9)	70.7 (35.1) * p-val= 0.0025
ESMO classification of patients	Intermediate risk (n)	16	14
	High risk (n)	9	17

n.s.

Table S13. Baseline characteristics of the PCa patients included in the study.
